# Negligible senescence in naked mole rats may be a consequence of well-maintained splicing regulation

**DOI:** 10.1007/s11357-019-00150-7

**Published:** 2020-01-11

**Authors:** B. P. Lee, M. Smith, R. Buffenstein, L. W. Harries

**Affiliations:** 1 Institute of Biomedical and Clinical Sciences, University of Exeter Medical School, Barrack Road, Exeter, EX2 5DW UK; 2 Calico Life Sciences LLC, 1170 Veterans Blvd., South San Francisco, CA 94080 USA

**Keywords:** Naked mole-rat, *Heterocephalus glaber*, Ageing, Splicing factors, *hnRNPs*, *Srsfs*, Klotho, CDKN2A, Cognitive function, DNA repair

## Abstract

Naked mole-rats (NMRs) have amongst the longest lifespans relative to body size of any known, non-volant mammalian species. They also display an enhanced stress resistance phenotype, negligible senescence and very rarely are they burdened with chronic age-related diseases. Alternative splicing (AS) dysregulation is emerging as a potential driver of senescence and ageing. We hypothesised that the expression of splicing factors, important regulators of patterns of AS, may differ in NMRs when compared to other species with relatively shorter lifespans. We designed assays specific to NMR splicing regulatory factors and also to a panel of pre-selected brain-expressed genes known to demonstrate senescence-related alterations in AS in other species, and measured age-related changes in the transcript expression levels of these using embryonic and neonatal developmental stages through to extreme old age in NMR brain samples. We also compared splicing factor expression in both young mouse and NMR spleen and brain samples. Both NMR tissues showed approximately double the expression levels observed in tissues from similarly sized mice. Furthermore, contrary to observations in other species, following a brief period of labile expression in early life stages, adult NMR splicing factors and patterns of AS for functionally relevant brain genes remained remarkably stable for at least two decades. These findings are consistent with a model whereby the conservation of splicing regulation and stable patterns of AS may contribute to better molecular stress responses and the avoidance of senescence in NMRs, contributing to their exceptional lifespan and prolonged healthspan.

**Electronic supplementary material:**

The online version of this article (10.1007/s11357-019-00150-7) contains supplementary material, which is available to authorized users.

## Introduction

Naked mole-rats (NMRs; *Heterocephalus glaber*) are eusocial animals which live in subterranean colonies of up to 300 individuals with a single breeding female, in a manner analogous to bees or other social insects (Jarvis 1981; Brett 1991). Similarly sized rodents such as mice have a lifespan limited to 3–4 years, whereas NMRs typically survive to over 20 years of age, with a maximum recorded lifespan of 37 years in a still extant male. This is approximately 5-fold greater than that predicted from their small body size (Edrey et al. 2011). This extraordinary lifespan is accompanied by extended “healthspan”, with virtually no increase in Gompertzian mortality risk with age (Buffenstein et al. 2012; Ruby et al. 2018). Both fertility and neurogenesis persist for decades (Holmes 2016; Lewis and Buffenstein 2016a, b). NMRs are resistant to both spontaneous and experimentally induced cancer (Liang et al. 2010; Delaney et al. 2013; Deweerdt 2014). Moreover, NMRs demonstrate few age-related metabolic, cardiac or vascular changes (O'Connor et al. 2002; Csiszar et al. 2007; Buffenstein 2008; Grimes et al. 2014; Triplett et al. 2015a, b; Delaney et al. 2016). Indeed, considerable evidence suggests they maintain an overall youthful phenotype throughout their long lives (Buffenstein et al. 2019), for example they retain foetal haemoglobin throughout their lives, facilitating more efficient oxygen transport and clearly an evolved adaption to the hypoxic challenges of their colonial, subterranean habitation. Having resided in this harsh milieu since the early Miocene, they have evolved numerous adaptations to deal with the many conditions imposed by their subterranean habitation (e.g. hypercapnia, hypoxia, exposure to heavy metals, and plant secondary defence mechanisms) for review see (Lewis and Buffenstein 2016a, b). For example, NMRs can survive 18 min in pure nitrogen atmospheres and under these anoxic conditions switch from glucose-based anaerobic metabolism to that of fructose and appear to have several neuroprotective mechanisms to prevent neuronal death under hypoxia (Park et al. 2017; Munro et al. 2019). These long living, resilient animals therefore present an interesting paradox in ageing biology and an opportunity to examine fundamental mechanisms of ageing.

Several molecular explanations for the enhanced longevity and negligible levels of senescence (Buffenstein 2008) of NMRs have been proposed, most of which align to the established “hallmarks of ageing” (Lopez-Otin et al. 2013; Dammann 2017). NMRs have a low metabolic rate approximately 70% of that of a laboratory mouse (Yahav and Buffenstein 1991), enhanced expression of DNA repair genes (MacRae et al. 2015), resistance to oxidative stress (Lewis et al. 2013), well-maintained proteostasis (Perez et al. 2008; Pride et al. 2015), enhanced autophagy (Zhao et al. 2014), improved telomere maintenance (Evfratov et al. 2014), a stable epigenome (Tan et al. 2017), a well-maintained circulating and hepatic metabolome consistent with methionine and dietary restriction (Heinze et al. 2018; Lewis et al. 2018; Viltard et al. 2019) and concomitant high levels of cystathionine β-synthase (CBS) activation (Olecka et al. 2018), a novel immune system (Hilton et al. 2019), and show few signs of cellular senescence both in vitro (Zhao et al. 2018) and in vivo (Buffenstein 2008). The lack of senescence is of particular interest since targeted ablation of p16^INK-4a^ positive senescent cells in genetically altered mouse models is able to extend lifespan and bring about improvements in many of the features of old age (Baker et al. 2011; Baker et al. 2016; Baar et al. 2017; Baar et al. 2018; Baker and Petersen 2018). Senescent cells are thought to drive ageing phenotypes by a number of mechanisms, not least the secretion of the senescence-associated secretory phenotype (SASP) and transcriptional changes brought about through chromatin remodelling (van Deursen 2014). Senotherapeutic interventions in man are in their infancy, but have been reported to have some beneficial effects in patients with idiopathic pulmonary fibrosis or chronic kidney disease (Hickson et al. 2019; Justice et al. 2019).

Cellular senescence can arise because of failure in molecular stress response as may occur during oncogenic insults (de Magalhaes and Passos 2018) and during development. A key component of this is the ability to maintain an active and plastic transcriptome. In response to internal and external stimuli, cells must be able to effectively reprogram their gene expression (Guzikowski et al. 2019). Alternative splicing (AS), one mechanism by which genes can produce a variety of functionally different isoforms from a single coding unit, is a key component of transcriptomic plasticity, and is especially responsive to stress (Nevo et al. 2012; Pai and Luca 2019). Unlike constitutive splicing, which occurs for all multi-exon genes, AS brings about the expression of a repertoire of multiple isoforms, which may be temporally or spatially regulated, or differ in response to stimuli. Alternative isoforms commonly have variant or antagonistic functions (Stamm et al. 2005; Villate et al. 2008). AS events may comprise alternative (cassette exons) exons, alternative 5′ or 3′ splice site usage, intron inclusion or deletions of parts of exons. More than one of these phenomena may be active in any given gene (Pai and Luca 2019). AS decisions are made by the binding of serine-arginine rich (SR) splicing activator proteins (SRSFs) and heterogeneous nuclear ribonucleoprotein particle (hnRNP) splicing inhibitor proteins to exon and intron splicing enhancer or silencer sequences around the splice sites (Cartegni et al. 2002). Each splice site is regulated by the combinational and competitive binding of a unique combination of activators and inhibitors (Smith and Valcarcel 2000). These activator and inhibitor proteins are collectively termed splicing factors. AS has previously been reported in NMRs, as in other higher organisms, including for important senescence-associated loci such as *CDKN2A* (Tian et al. 2015).

Correct regulation of AS is critical to ageing; genes encoding components of the splicing regulatory machinery are amongst the most dysregulated by age in human populations and in senescent human fibroblasts, endothelial cells, cardiomyocytes and astrocytes. Splicing factor expression is mainly downregulated in senescent cells of these subtypes, but tissue-specific differences in both the identity of affected splicing factors and directionality do exist (Harries et al. 2011; Holly et al. 2013; Latorre et al. 2017; Latorre et al. 2018a; Latorre et al. 2018b; Lye et al. 2019). The expression of splicing regulatory factor genes are also associated with lifespan and dietary restriction in mice and other species (Heintz et al. 2016; Lee et al. 2016; Lee et al. 2019a), and predictively linked with human ageing phenotypes in population studies (Lee et al. 2019b; Lye et al. 2019). Splicing factors expression is tightly connected to control of cell proliferation with splicing factors frequently mutated in cancer (Seiler et al. 2018). Finally, restoration of splicing factor expression using small molecules or targeted genetic interventions is able to reverse multiple features of senescence in aged human primary cells in vitro (Latorre et al. 2017; Latorre et al. 2018a, c).

We hypothesised that given the lack of visible signs of senescence in ageing NMRs and the importance of splicing factor regulation in the context of senescence, that splicing factor dysregulation and the consequent changes to the splicing patterns in ageing cells and tissues (Harries et al. 2011; Latorre et al. 2018b; Lye et al. 2019) may not be a feature of NMR ageing. We aimed to characterise the abundance of an a priori panel of 20 splicing factors known to be important in ageing and senescence from our previous work (Holly et al. 2013; Latorre et al. 2017; Latorre et al. 2018a, b, c), in a series of whole brain samples originating from embryonic NMRs all the way up to extreme old age. Brain expression levels of a panel of senescence-related genes, and AS patterns of a candidate series of functionally relevant brain isoforms previously identified to be altered in replicatively senescent human astrocytes (Lye et al. 2019) were also assessed as a functional output of splicing factor expression. We determined that although changes are evident in the brain expression of splicing factor genes between embryonic and adult states as expected, splicing factor expression trajectories remain static over the course of NMR ageing, as do the expression patterns of important alternatively spliced brain function genes. Furthermore we identified no increase in the expression of important molecular markers of cellular senescence (including isoforms of the *Cdkn1a Tp53* and *Cdkn2a* genes*)*, in accordance with the negligible senescence phenotype in these animals. Our data are supportive of a hypothesis that the extraordinary longevity of NMRs may arise at least in part from maintained transcriptomic molecular stress responses, conservation of regulated alternative splicing leading to avoidance of cellular senescence, a post-transcription mechanism of gene expression regulation.

## Methods

### Animal characteristics and husbandry

The NMRs used in this study were part of the well characterised Calico colony. The progenitors of these animals were collected in Kenya. All animals are microchipped at 90 days of age, providing each individual with a unique nine-digit identifying number. NMRs were housed in multi-chambered plexiglass burrow systems in animal rooms maintained at 28–30 °C and 30–50% relative humidity, in attempts to simulate climatic conditions in their native equatorial habitat. The animals were fed ad libitum with fruit and vegetables (bananas, apples, oranges, butternut squash, red bell pepper, romaine lettuce, cucumber, green beans, corn, carrots and red garnet yams) and supplemented with a high protein and vitamin enriched cereal (Pronutro, South Africa).

Animals, ranging from neonates to 22 years old, as well as pregnant females were euthanised using isoflurane and killed by cardiac exsanguination. Four to eight animals of both sexes were used in each age cohort (Table 1). Tissues were harvested from the embryos and animals of different ages and immediately flash frozen in liquid nitrogen and stored at -80 °C until analysed. Brain and spleen samples were used in these studies. We chose spleen because, as a lymphoid organ, it consists of large numbers of white blood cells. Most of the transcripts extracted from spleen will arise from B cells, T cells and mononuclear phagocytes and may allow a window on “inflammaging”, thought to be a prominent ageing mechanism (Zuo et al. 2019). Brain was chosen as splicing patterns are especially complex and functionally important in this organ (Karlsson and Linnarsson 2017), and dysregulated splicing is a feature of many neurodegenerative and neurodevelopmental diseases in brain (Nik and Bowman 2019). All animal use and experiments were approved by the Buck Institute Institutional Animal Care and Use Committee (IACUC) protocol number A10173.

**Table 1:** Details of animals used in the study. Shown here are the numbers and age ranges of naked mole rats included in each age category in the current study

Sample subset	Age range (days)	*n*
Foetus	–	5
1 day	1	6
2 weeks	14	4
3–4 years	1095–1793	8
5–6 years	2163–2478	4
7–8 Years	2631–2920	4
9–12 years	3306–4264	6
13–15 years	4745–5326	5
17–20 years	6264–7300	5
21–22 years	7842–8159	4

### Candidate genes selected for analysis

An a priori list of splicing factor candidate genes were chosen based on associations with ageing or senescence in multiple human ageing cohorts and in senescent primary human cell lines (Harries et al. 2011; Holly et al. 2013; Latorre et al. 2017; Latorre et al. 2018b). Some of the splicing factors in this list have also been shown to associate with lifespan in both mice and humans (Lee et al. 2016) and cognitive dysfunction in human populations (Lee et al. 2019b), or to be involved in molecular responses to dietary restriction (Lee et al. 2019a). The list of genes included the negative regulatory splicing factors *Hnrnpa0*, *Hnrnpa1*, *Hnrnpa2b1*, *Hnrnpd*, *Hnrnph3*, *Hnrnpk*, *Hnrnpm*, *Hnrnpul2*, the positive regulatory splicing enhancers *Pnisr*, *Srsf1*, *Srsf2*, *Srsf3*, *Srsf6*, *Tra2b* and the core components of the spliceosome *Sf1* and *Sf3b1*. A further panel of alternatively spliced candidate genes where specific isoforms have known links to brain function/dysfunction (Lye et al. 2019) and/or have been demonstrated to be dysregulated in senescent astrocytes (Lye et al. 2019) or linked with cognitive dysfunction in human populations (Lye et al. 2019), and/or neurodegenerative disease were chosen for analysis. These were *Aph1a*, *App*, *Aqp4*, *Gfap*, *Klotho, Mapt, Psen1* and *Psen2.* An additional panel of transcripts known to be associated with senescence were also used: *Atm*, *Cdkn1a*, *Cdkn2a*, *Cdkn2*b and *Tp53*. Table 2 lists all genes/isoforms tested in this panel, rationale for their inclusion and references to supporting literature. Isoform structures are given in Supplementary figure S1. Six endogenous control genes were also selected on the basis of stability in ageing human populations (Harries et al. 2011): *Hprt1*, *Idh3b*, *Polr2a*, *Ppia*, *Tbp* and *Ywhaz*, however assays to *Hprt1* and *Ywhaz* were subsequently excluded due to poor assay performance.

**Table 2:** Cognition and senescence-related transcripts and isoforms used for expression analysis. Shown here are the transcripts and isoforms selected for analysis and a brief description of their function. Details of the type of AS event for each isoform, and the exons involved are provided in the references indicated

	Gene	Transcripts/isoforms	Function	References
Cognition-related transcripts	*Aph1a*	*Aph1aL*	Subunit of the gamma-secretase complex involved in β-amyloid processing	(Ma et al. 2005), (Serneels et al. 2005)
		*Aph1aS*		
	*App*	*App*_*695*_	Precursor to β-amyloid protein	(Rohan de Silva et al. 1997), (Zhang et al. 2011)
		*App*_*714*_		
		*App*_*751*_		
		*App*_*ALL*_		
	*Aqp4*	*Aqp4-M1*	Pore-forming intrinsic membrane protein	(De Bellis et al. 2014), (Jung et al. 1994), (Mader and Brimberg 2019)
		*Aqp4-M23*		
	*Gfap*	*Gfapα*	Astrocyte intermediate filament protein	(Kamphuis et al. 2012)
		*Gfapδ*		
	*Mapt*	*Mapt-3R*	Microtubule-associated protein tau involved in neurofibrillary tangles	(Goedert et al. 1989), (Lacovich et al. 2017)
		*Mapt-4R*		
	*Klotho*	*mKlotho*	Endocrine factor which improves cognitive performance in ageing	(Masso et al. 2015)
		*sKlotho*		
	*Psen1*	*Psen1*	Subunit of the gamma-secretase complex involved in β-amyloid processing	(De Jonghe et al. 1999), (Janssen et al. 2000)
		*Psen1 (ins*_*TAC*_*)*		
		*Psen1 (VRSQ)*		
	*Psen2*	*Psen2*	Subunit of the gamma-secretase complex involved in β-amyloid processing	(Moussavi Nik et al. 2015), (Sato et al. 1999)
		*Psen2 (Δexon5)*		
Senescence-related transcripts	*Atm*	*Atm*	DNA damage repair	(Marechal and Zou 2013)
	*Cdkn1a*	*Cdkn1a (p21a)*	Inhibitory to proliferation	(Kaija et al. 2012), (Nozell and Chen 2002)
	*Cdkn2a*	*Cdkn2a (p14*^*ARF*^*)*	p53 pathway to cell cycle arrest	(Tian et al. 2015), (Kim et al. 2011)
		*Cdkn2a (p16*^*INK4a*^*)*	RB1 pathway to cell cycle arrest	
	*Cdkn2b*	*Cdkn2b (p15*^*INK4b*^*)*	TGFβ pathway to cell cycle arrest	(Tian et al. 2015)
	*Tp53*	*Tp53*	Cell cycle regulation	(Vousden and Lane 2007)

### RNA extraction

Snap-frozen tissues were first treated with RNAlater™-ICE Frozen Tissue Transition Solution (ThermoFisher, Waltham, MA, USA) according to the manufacturer’s instructions, to allow handling of tissue without RNA degradation occurring due to thawing of sample. Tissue sections were placed in 1 mL TRI Reagent® Solution (ThermoFisher, Waltham, MA, USA) supplemented with the addition of 10 mM MgCl_2_ to aid recovery of microRNAs (Kim et al. 2012). Samples were completely homogenised in a bead mill (Retsch Technology GmbH, Haan, Germany) at a frequency of 30 cycles per second for 15 min. Phase separation was carried out using chloroform. Total RNA was precipitated from the aqueous phase by means of an overnight incubation at − 20 °C with isopropanol. A total of 1.2 μl Invitrogen™ GlycoBlue™ Coprecipitant (ThermoFisher, Waltham, MA, USA) was added prior to incubation to aid pellet recovery. RNA pellets were ethanol-washed twice and re-suspended in 1× TE buffer, pH 8.0. RNA quality and concentration were assessed by NanoDrop spectrophotometry (NanoDrop, Wilmington, DE, USA).

### Reverse transcription

For RTPCR and Sanger sequencing as described below, a pool of 1000 ng of total RNA taken from 10 samples (one sample chosen at random from each NMR age group) was reverse transcribed using EvoScript Universal cDNA Master kit (Roche LifeScience, Burgess Hill, West Sussex, UK) in single 20 μl reactions, according to the manufacturer’s instructions, with the exception of a change to the extension phase of the reaction: a step of 30 min at 65 °C was used instead of 15 min at 65 °C. Resulting cDNA samples were diluted to a final volume of 75 μl with dH_2_O, and repeated as necessary to provide sufficient template for all RTPCR and sequencing reactions carried out. For assay validation by standard curve, reactions as described above were carried out in quadruplicate and pooled prior to a 6-step 1:2 dilution series. For qPCR, 1500 ng of total RNA was reverse transcribed using SuperScript® VILO™ cDNA Synthesis Kit (ThermoFisher, Waltham, MA, USA) in 20 μl reactions, according to the manufacturer’s instructions (Thermofisher n.d.a, b). Resulting cDNA samples were diluted to a final volume of 150 μl with dH_2_O to ensure sufficient volume for all subsequent qPCR reactions.

### Assay design

Quantitative real-time reverse transcriptase PCR (qPCR) assays to mouse splicing factor assays were obtained from Thermo Fisher (ThermoFisher, Waltham, MA, USA). Assay Ids are available on request. Assays to NMR splicing factors and alternatively expressed isoforms were custom designed to determine expression levels of all candidate mRNAs. Given that the current NMR genome is not completely annotated, this required a four-step design process, as follows:

- “In silico” sequence alignment: Where genes were not being tested for alternate isoform expression, the genome sequences of genes in question were obtained from the UCSC Genome Browser (Kent et al. 2002) for the following species: NMR, Guinea pig, Rat, Mouse and Human (see Supplementary Table S1 for assemblies used). Intronic sequences were removed and resulting “in silico-spliced” sequences manually aligned across all 5 species to identify areas of highest homology. Where feasible, exon boundaries within the regions of high homology were then selected as target areas for design of qPCR assays. Where alternate isoforms exist and were required to be assessed, evidence from previous literature was sought to identify the isoforms in question, with particular focus on differential function of specific isoforms (the nature of each splicing change and the precise exons involved are shown in Supplementary Figure S1, and referenced in Table 2). Dependent upon the species in which previous work had been carried out, one or more sequences were obtained from the UCSC Genome Browser (Kent et al. 2002) along with the NMR sequence. Once again, “in silico splicing” and manual alignment was carried out as described in the previous paragraph, however in these cases the areas of alternative splicing were selected as targets for assay design.
- Validation of exon junction sequences: Prior to design of qPCR assays, sequence verification was carried out by conventional RTPCR amplification of the predicted fragments to confirm identity. In all cases, primers were designed to the NMR sequence, with the aim of amplifying the target areas as defined above and confirming amplicon identity based on expected size. Where alternative spliced isoforms were to be amplified (and where isoform structure allowed) multiple primer sets were designed to amplify each isoform individually. In the cases of *Aph1a*, *App* and *Mapt*, this was not possible therefore one set of primers was designed to amplify all possible isoforms along with a set of nested primers to isolate individual isoforms using gel electrophoresis and band-stab PCR (Harries et al. 2004). Primer sequences are given in Supplementary Table S2. Template cDNA for the RTPCR was created from a pool of mRNA from all NMR age groups. This pool was reverse transcribed as described above and used in PCR reactions using either Microzone Megamix Royal (Clent Life Science, Stourbridge, UK) or Platinum™ II Hot-Start Green PCR Master Mix (ThermoFisher, Waltham, MA, USA). Reaction conditions were set according to manufacturer’s instructions except in some cases where annealing temperatures and cycle numbers used were specific to each amplicon. These are given in Supplementary Table S2. Amplicons were then checked using gel electrophoresis, and those which matched the expected size were taken forward for the next steps. Of the genes chosen, *App* and the p21b isoform of *Cdkn1a* proved refractory to RTPCR amplification and size-based verification. In the case of *App*, the predicted sequence was used for the assay design. For p21b, sequence homology between the known (human/mouse) variants and the NMR genome assembly was too low to design an assay with any degree of confidence, so this isoform was omitted at this stage.
- Sanger sequencing: Once amplicons had been verified by size, each was then sequenced to absolutely confirm the in silico-predicted spliced mRNA sequence. Sanger sequencing was carried out on the ABI 3730 platform using standard protocols. PCR primers were designed to incorporate M13 forward and reverse tag sequences to enable use of a common sequencing primer for all reactions (see Supplementary Table S2). Sequence verification was successful in all cases, however the NMR *Psen1* sequence showed no evidence of either the 3 base insertion (ins_TAC_) or the 4 amino acid (VRSQ) splice variants previously reported in human studies (De Jonghe et al. 1999; Janssen et al. 2000), while the NMR *Psen2* sequence did not contain the exon 5 skipped (PS2V) splice variant seen in human and animal studies (Sato et al. 1999; Moussavi Nik et al. 2015). Consequently, both genes were omitted from further analysis.
- Assay design and verification: Following sequence confirmation, TaqMan® assays were designed to target regions as defined above. Forward primer, reverse primer and reporter sequences of all assays designed are given in Supplementary Table S3. To determine assay efficiency and linearity, standard curves were created from qPCR data generated using a serial dilution of NMR cDNA (see “Reverse transcription” section above). Assay efficiencies and *r*^2^ values are given in Supplementary Table S3.

### Quantitative reverse transcriptase real-time PCR

A total of 1.0 μl cDNA (reverse transcribed as indicated above) was added to a 5 μl qRTPCR reaction including 2.5 μl TaqMan® Universal Master Mix II, no UNG (ThermoFisher, Waltham, MA, USA) and 0.25 μl custom TaqMan® probe and primer mix (corresponding to 900 nM each primer and 250 nM probe). Reactions were run in triplicate on 384-well plates using the QuantStudio 12 K Flex Real-Time PCR System (ThermoFisher, Waltham, MA, USA). Amplification conditions were a single cycle of 95 °C for 10 min followed by 40 cycles of 95 °C for 15 s and 60 °C for 1 min. We first compared levels of splicing factor transcripts in spleen and brain tissue from young NMR (12 months) and young mice (3–4 months), followed by assessment of splicing factor expression and alternative splicing patterns of key senescence or brain-related genes in an extended series of NMR brain tissues from foetal stages to extreme old age (21 to 22 years).

### Data preparation

EDS files were uploaded to the ThermoFisher Cloud (ThermoFisher, Waltham, MA, USA) and analysed using the Relative Quantification qPCR App within the software (ThermoFisher^a^). To ensure we are looking at real difference and not efficiency differences, assays were designed to the same splice boundary and are as conserved as possible. Relative threshold data were calculated using the C_rt_ method employed by the ThermoFisher analytical software (ThermoFisher^b^) along with correction for assay efficiencies (Supplementary Table S3) to produce relative threshold data and then imported into Excel (Microsoft, Redmond, WA, USA) and used for analysis using the 2^−ΔΔCrt^ method. Separate analyses were carried out for the splicing factor species comparison, splicing factor brain series and the isoform/senescence brain series datasets. First, C_rt_ data from all transcripts measured, housekeeping endogenous controls, calculated arithmetic and geometric means of these controls along with calculated “global” arithmetic and geometric means across all genes measured for each dataset were then uploaded to the RefFinder webtool (Xie et al. 2012) to establish the most stable transcript(s). For both the species comparison and the splicing factor brain series, the geometric mean of the 4 housekeeping endogenous controls (*Idh3b*, *Ppia*, *Polr2a* and *Tbp*) was most stable, while in the isoform/senescence brain series the geometric mean across all genes measured was the most stable, thus these were used for the respective ΔC_rt_ normalisation steps. For the ΔΔCrt normalisation, in the case of the species comparison the arithmetic mean expression value of all transcripts across all mouse samples from both tissues was used as the comparator. In the splicing factor brain series, the arithmetic mean expression level in the 3–4 year old animals for each transcript was used for ΔΔCrt normalisation, as this represents a mature (i.e. non-developmental), but comparatively young time point for comparison. For the alternatively spliced isoform/senescence brain series, the arithmetic mean of the expression levels in the 3–4-year-old animals for each isoform was used (as above) for the statistical analyses as this enabled us to test expression differences between time points longitudinally for each isoform, however for visualisation purposes (see Figs. 4 and 5) and to allow for illustration of the relative expression levels of each isoform, a separate analysis was carried out using a median value across all isoforms for the ΔΔCrt normalisation. Following these ΔΔC_rt_ normalisation steps, fold-changes were calculated using the 2^−ΔΔCrt^ method. To ensure normal distribution and for ease of visualisation, data were subsequently log transformed; in the case of the species comparison, a log_10_ transformation was used due to the effect sizes, while for the splicing factor brain series and isoform/senescence brain series, a natural log was employed.

## Statistical analysis

Differences in gene expression were tested using Student’s *t* test for the species comparison dataset and linear regressions for both the splicing factor brain series and isoform/senescence brain series datasets. Student’s *t* test were carried out in SPSS v15.0 (IBM, Armonk, NY, USA) and linear regressions in STATA v15.1 (StataCorp, College Station, TX, USA). Benjamini, Krieger and Yekutieli false discovery rate (FDR) calculations (Benjamini et al. 2006) were then performed to account for multiple testing using GraphPad Prism 8.2.0 (GraphPad Software, San Diego, CA, USA), with the *q* value set at 1%.

## Results

### Splicing factors are generally expressed at higher levels in young NMR tissues compared to young mouse tissues

**Fig. 1:**
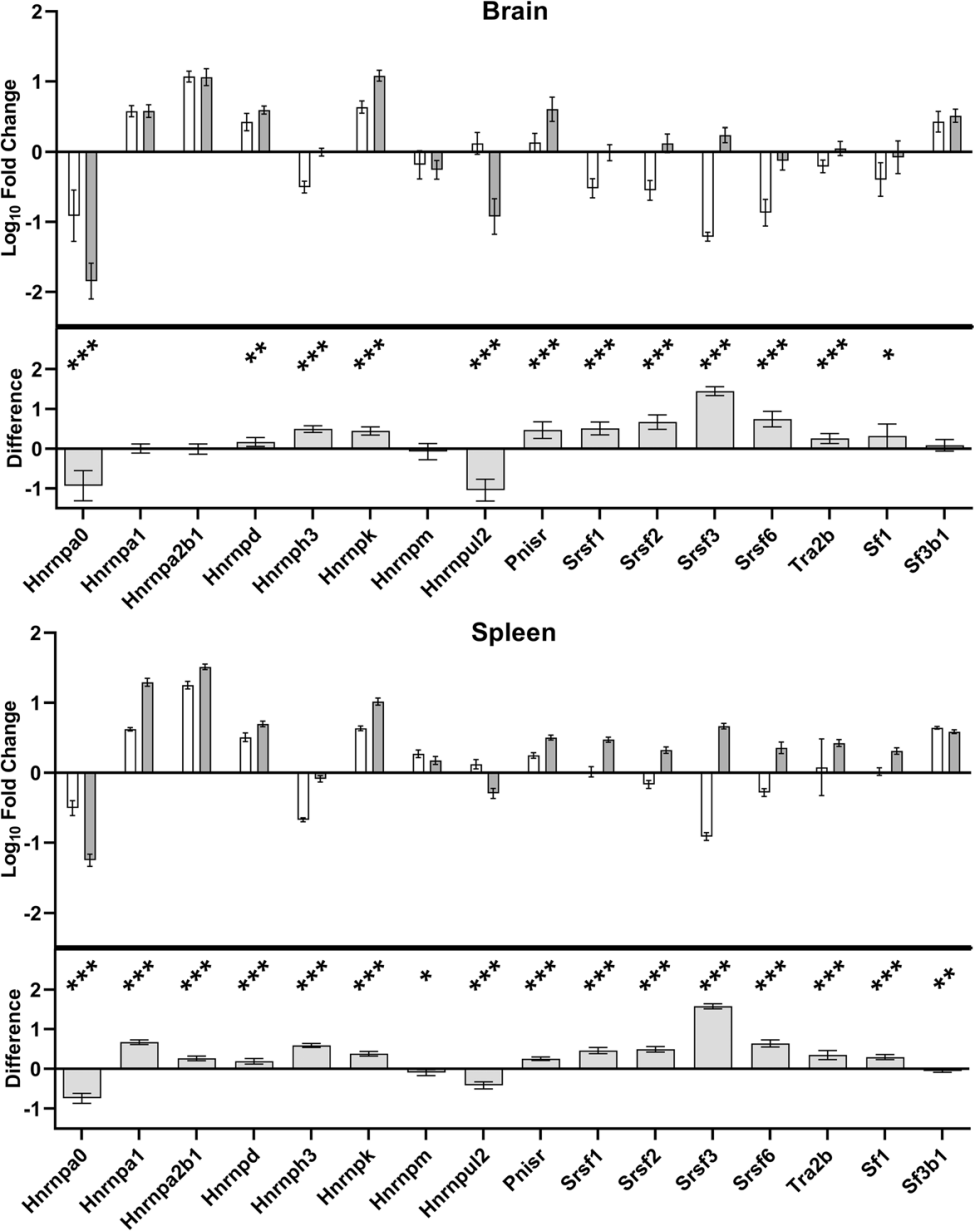
Comparison of splicing factor transcript expression levels between mouse and naked mole-rat (NMR) in brain and spleen. The upper panel of each graph shows log_10_ fold-change in expression levels, relative to the mean expression across all transcripts in both tissues in mice, with open bars denoting expression in mouse and grey bars showing expression in the NMR. The lower panel of each graph shows the difference between the species. Significant differences between species are denoted with stars: * = *p* < 0.05, ** = *p* < 0.01, *** = *p* < 0.001. All significant differences noted on the graphs met the FDR correction criteria at *p* < 0.046.

We compared splicing factor expression in the spleen and brain of young mice and naked mole-rats. We used spleen and brain because of the known role of inflammation in the ageing process and the known complexity and prevalence in age-related diseases linked to brain splicing patterns. Looking at splicing factor expression as a whole, relative levels across all transcripts measured are on average 1.6× and 2.1× higher in young NMR brain and spleen respectively compared with young mice. In spleen, all splicing factors tested demonstrated altered expression in NMRs compared with mice. Most genes were upregulated. In brain, although slightly fewer splicing factors demonstrated altered expression compared to mice, major differences in expression were apparent, particularly for the *Hnrnpa0*, *Hnrnph3, Hnrnpk, Hnrnpul2, Srsf1, Srsf2, Srsf3* and *Srsf6* genes (*p* = < 0.001 for each). There was a striking similarity in the overall pattern of species differences in splicing factor expression in both tissues, with 13 out of the 16 splicing factors measured sharing directionality and approximate effect size across the two tissues (Fig. 1, Supplementary Table S4).

### Splicing factor transcript expression remains stable during NMR ageing

**Fig. 2:**
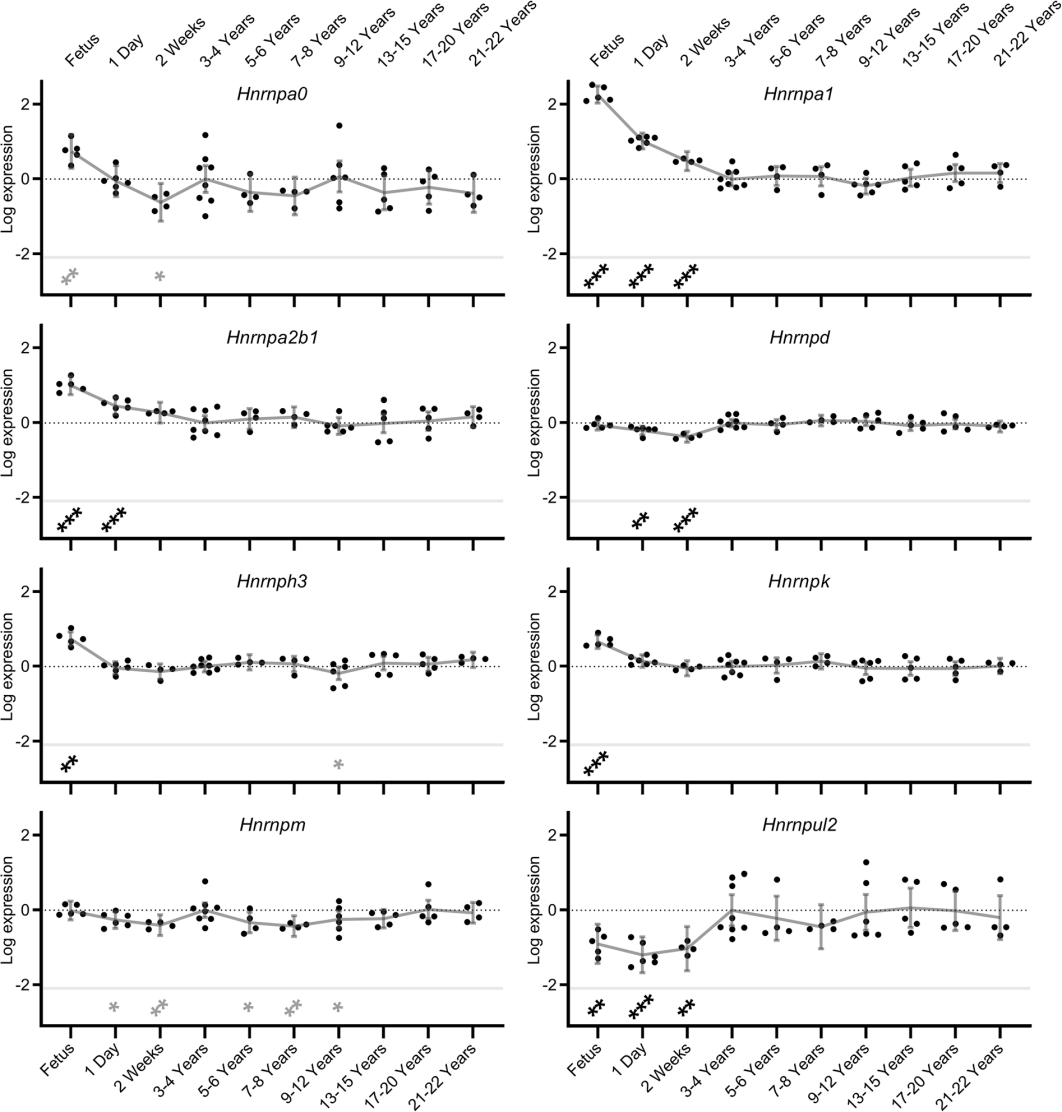
*S*plicing inhibitor expression levels in naked mole-rat (NMR) brain during development and ageing. Transcript expression levels of Hnrnp splicing inhibitors in NMR brain from foetus through to 20–22 years old are shown. Data points represent β-coefficients of log fold-change in expression levels for each transcript in each animal relative to the mean expression in the 3–4 year old animals. The null point for each transcript is shown as a light dotted line. Grey lines denote the mean values with error bars at the 95% confidence intervals. Transcripts showing significant differences from the 3–4 year old comparator group are denoted with stars: * = *p* < 0.05, ** = *p* < 0.01, *** = *p* < 0.001. Associations which remain significant after FDR correction (*p* < 0.002) are indicated in black, while those in grey represent nominal associations (*p* < 0.05).

**Fig. 3:**
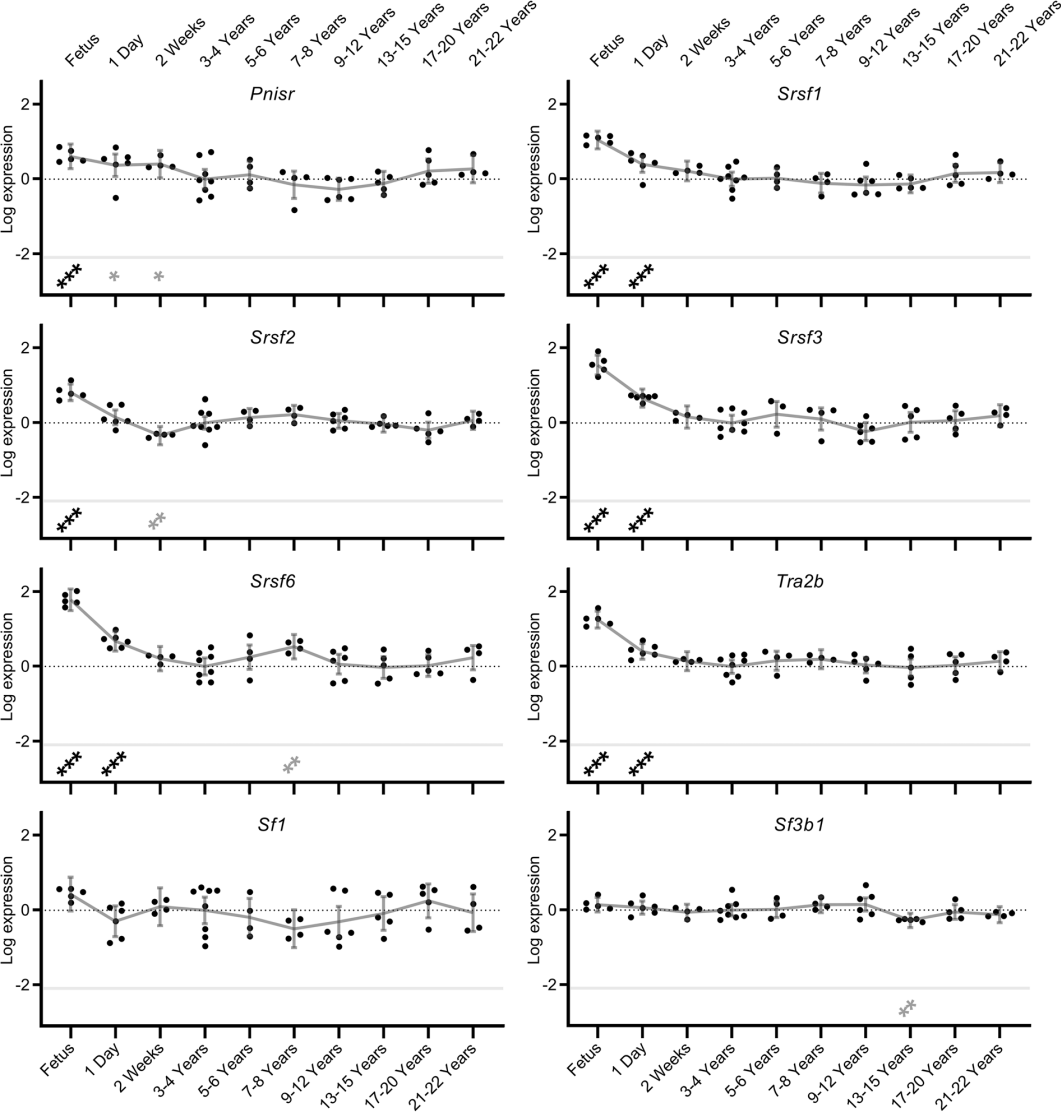
Splicing activator and core spliceosome component expression levels in naked mole-rat brain during development and ageing. Transcript expression levels of Srsf splicing activators in NMR brain from foetus through to 20–22 years old are shown. Data points represent β-coefficients of log fold-change in expression levels for each transcript in each animal relative to the mean expression in the 3–4-year-old animals. The null point for each transcript is shown as a light dotted line. Grey lines denote the mean values with error bars at the 95% confidence intervals. Transcripts showing significant differences from the 3–4 year old comparator group are denoted with stars: * = *p* < 0.05, ** = *p* < 0.01, *** = *p* < 0.001. Associations which remain significant after FDR correction (*p* < 0.002) are indicated in black, while those in grey represent nominal associations (*p* < 0.05).

To investigate changes in splicing factor expression across the lifecourse, splicing factor expression was measured from developmental stages (foetus, 1-day-old or 2-week-old animals) until old age (21–22 years). For all regulatory splicing factor transcripts measured, significant expression changes were noted in the brain tissue during at least one of the developmental phases (foetus, 1-day-old or 2-week-old). Once the NMRs were older than 2 weeks of age, for both splicing activators and inhibitor transcripts, in most cases splicing factor expression declined markedly (*p* < 0.001) and stabilised at these low levels for more than two decades. This was most marked for *Hnrnpa1*, *Hnrnpa2b1*, *Hnrnph3* and *Hnrnpk* splicing inhibitors (beta coefficients of expression change between foetal and 3/4 years were: 2.264, 0.997, 0.743 and 0.664 respectively; all *p* = <0.001) and *Srsf1, Srsf2, Srsf3, Srsf6* and *Tra2b* splicing activators (beta coefficients: 1.050, 0.812, 1.541, 1.786 and 1.257 respectively; all *p* = <0.001; Figs. 2 and 3; Supplementary Table S5). However in the case of *Hnrnpul2*, levels were lower in developmental states (up to 2 weeks of age) and were higher in the older age cohorts (beta coefficient: − 0.898; *p* = 0.001). During progression from mature adults (3–4 years old) to old age (21 to 22 years), almost no alterations in splicing factor expression were noted. Only 3 of 16 regulatory splicing factors (*Hnrnph3*, *Hnrnpm and Srsf6*) showed slight alterations in splicing factor expression during ageing in comparison to the 3–4-year-old animals (beta coefficients: − 0.181, − 0.245 and 0.529 respectively *p* = 0.036, 0.036 and 0.002; Figs. 2 and 3, Supplementary Table S5). Similarly, the core spliceosome element *Sf3b1* did not show any alterations in brain expression levels either during developmental phases or during ageing, with the exception of a single nominally significant reduction in expression of *Sf3b1* in the 13–15-year-old group (beta coefficient: − 0.275; *p* = 0.006; Supplementary table S6). This is contrary to previous observations from our group in other species, where dysregulation of splicing factor expression was noted in senescent astrocytes (Supplementary figure S2a; data from Lye et al. 2019).

### Cognition-related alternatively spliced isoform transcripts remain stable during NMR ageing

**Fig. 4:**
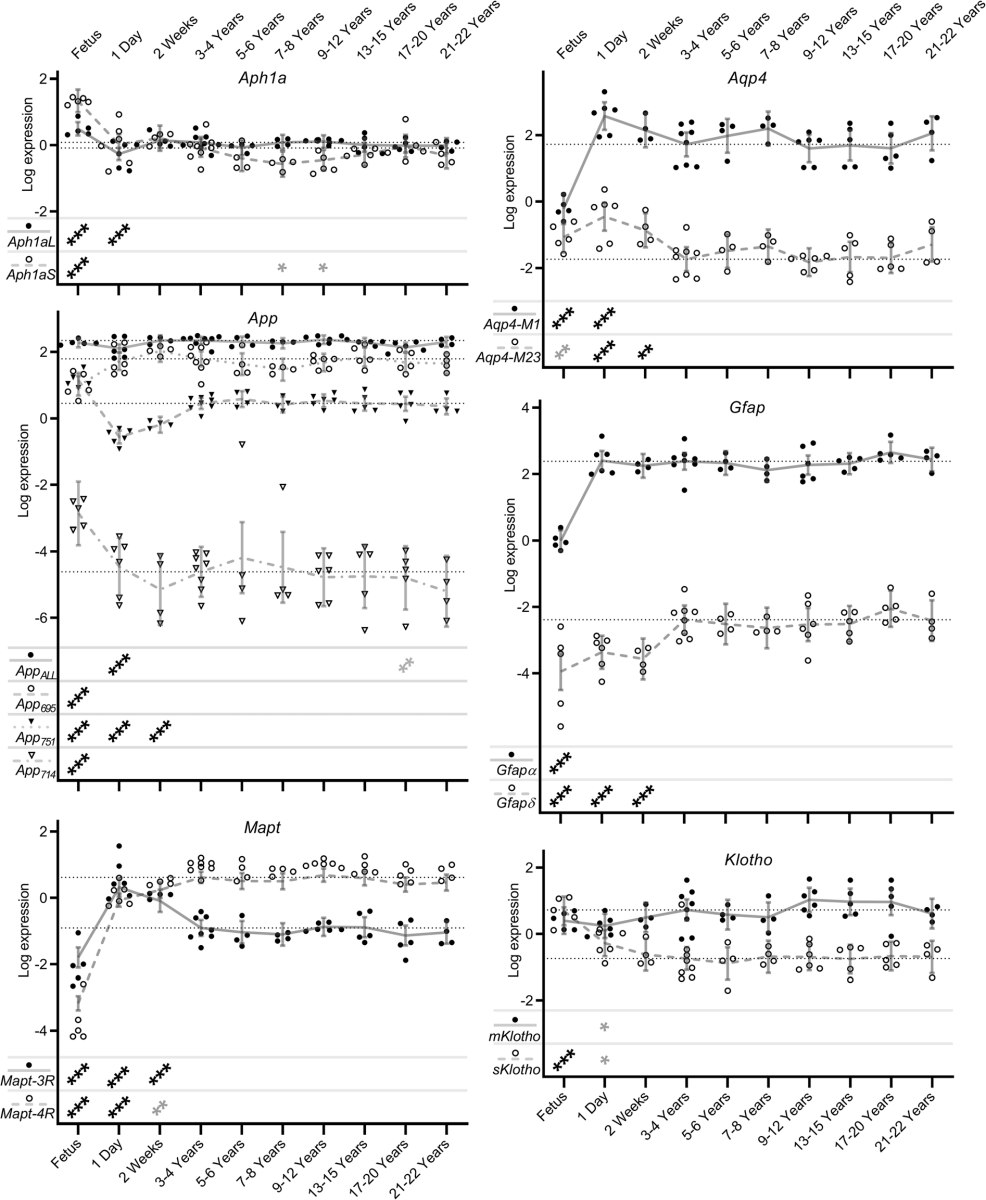
Cognition-related alternatively spliced isoform transcript expression levels in naked mole-rat brain during development and ageing. Shown here are transcript expression levels of cognition-related alternatively spliced isoform transcripts in NMR brain during development and ageing from foetus through to 20–22 years old. In order to show levels of individual isoform transcripts, expression levels are plotted relative to the median expression value of all isoforms across all time points within each gene. Data points represent β-coefficients of log fold-change in expression levels for each transcript in each animal relative to the mean expression of each isoform in the 3–4 year old animals (null point for each isoform shown as a light dotted line). Grey lines denote the mean values with error bars at the 95% confidence intervals. Isoform identities are shown in the legend at the lower left of each graph. Transcripts showing significant differences from the 3–4 year old comparator group are denoted with stars: * = *p* < 0.05, ** = *p* < 0.01, *** = *p* < 0.001. Associations which remain significant after FDR correction (*p* < 0.002) are indicated in black, while those in grey represent nominal associations (*p* < 0.05).

To demonstrate the functional consequences of splicing factor changes during NMR development and ageing, we assessed changes in the isoform repertoire of a panel of genes that we have previously demonstrated to be differentially expressed in human astrocyte senescence; some of which are also associated with cognitive decline in human populations (Lye et al. 2019). Sanger sequencing of the relevant isoform-specific exon boundaries demonstrated that the majority of the isoforms selected for assessment were also present in NMR. This included the p14^Arf^, p15 and p16^INK-4a^ isoforms of the *CDKN2A* gene; the mole-rat p16^INK-4a^ transcript has poor sequence similarity to that of the mouse, and reportedly presents with two early stop codons, predicting a truncated protein and while the functional domains appear to be partially preserved, the function of this cellular senescence pathway may be already compromised (Kim et al. 2011). The only cases where we did not find evidence for a NMR isoform corresponding to the human and mouse isoforms was the p21b isoform of *Cdkn1a.* Similar to the pattern seen in the splicing factors, we identified few changes in the expression levels of specific isoforms of cognition-related genes in adult animals. Levels of *Aph1a* isoforms demonstrated elevated expression in the foetal period (beta coefficients: 0.411 and 1.423, both *p* = <0.001; Fig. 4; Supplementary table S6) as did the expression of 2 of the 4 isoforms (App_714_ and App_751_) expressed from the *App* gene (beta coefficients: 1.758, and 0.699; both *p* = <0.001; Fig. 4; Supplementary table S6). Conversely one *App* isoform, App_695_ demonstrated decreased expression in the foetal period (beta coefficient: − 0.809; *p* = <0.001). Similarly, levels of both isoforms of *Mapt*, both isoforms of *Gfap* and the M1 isoform of *Aqp4* also demonstrated decreased expression in the foetal period (beta coefficients: − 1.096, − 4.661, − 2.393, − 1.563 and − 1.941; all *p* = <0.001; Fig. 4; Supplementary table S6). In most cases, isoforms were regulated independently of other isoforms of the same gene, indicating that these represent splicing changes and not changes in pre-mRNA transcription per se. Conversely, few changes were noted after 3–4 years old, with isoform ratios remaining consistent throughout the NMR ageing process. (Fig. 4; Supplementary table S6). This is contrary to observations from our group in other species, where dysregulation of this isoform set was reported in senescent astrocytes (supplementary figure S2b; data from Lye et al. 2019).

### Senescence-related transcripts remain stable during NMR ageing

**Fig. 5:**
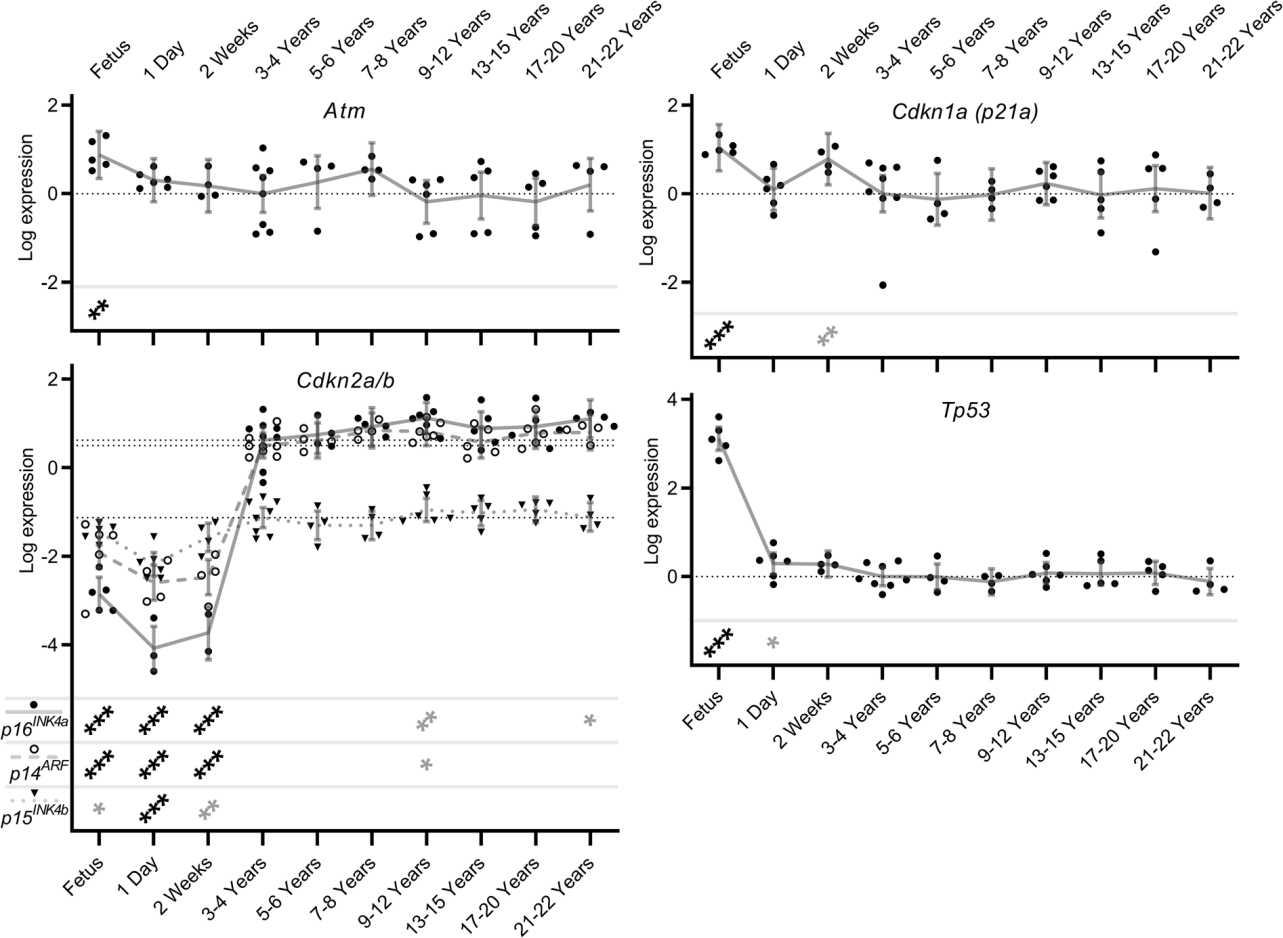
Senescence-related alternatively spliced isoform transcript expression levels in naked mole-rat brain during development and ageing. Shown here are transcript expression levels of senescence-related alternatively spliced isoform transcripts in NMR brain during development and ageing from foetus through to 20–22 years old. In order to show levels of individual isoform transcripts, expression levels are plotted relative to the median expression value of all isoforms across all time points within each gene. Data points represent β-coefficients of log fold-change in expression levels for each transcript in each animal relative to the mean expression of each isoform in the 3–4-year-old animals (null point for each isoform shown as a light dotted line). Grey lines denote the mean values with error bars at the 95% confidence intervals. Where applicable, isoform identities are shown in the legend at the lower left of the graph. Transcripts showing significant differences from the 3–4 year old comparator group are denoted with stars: * = *p* < 0.05, ** = *p* < 0.01, *** = *p* < 0.001. Associations which remain significant after FDR correction (*p* < 0.002) are indicated in black, while those in grey represent nominal associations (*p* < 0.05).

The negligible senescence noted in NMR (Buffenstein 2008; Stenvinkel and Shiels 2019) led us to evaluate the expression of known markers of senescence across the lifecourse in our samples. As seen for the splicing factor genes, expression levels of *Atm*, *Cdkn1a*, and *Tp53* genes were elevated in the foetal state (beta coefficients: 0.883, 1.040 and 3.111; *p* = 0.002, < 0.001 and < 0.001 respectively; Fig. 5; Supplementary table S7). Conversely, all 3 isoforms of the *Cdkn2a/b* locus (p14^ARF^, p16^INK4a^ and p15^INK4b^) demonstrated reduced expression in foetal animals compared with the mature animals (beta coefficients − 2.414, − 3.472 and − 0.336; *p* = <0.001, < 0.001 and 0.024 respectively; Fig. 5; Supplementary table S7). Subsequent trajectory for all the molecular markers of senescence remained remarkably static from young adult to old age (Fig. 5; Supplementary Table S7). This is in striking contrast to our observations in replicatively senescent human astrocytes, where marked dysregulation of this isoform set was observed (Supplementary Figure S1b; data from Lye et al. 2019).

## Discussion

Here we present evidence that, contrary to observations in both man and mouse, and more in keeping with their negligible senescence phenotype (Buffenstein 2008), adult NMRs maintain splicing regulatory capacity and conservation of regulated alternative splicing with advancing age. Splicing factor expression is higher in young adult NMRs than in young adult mice. We have also identified that contrary to observations from ourselves and others in humans (Harries et al. 2011; Holly et al. 2013; Wang et al. 2018) and in human senescent cells of several different lineages (Holly et al. 2013; Latorre et al. 2017; Latorre et al. 2018b; Lye et al. 2019), splicing factor expression and patterns of isoform expression in NMRs do not appear to alter with age. Splicing factor expression has been demonstrated to correlate with median strain lifespan in mice (Lee et al. 2016), and to have potential mechanistic roles in response to dietary restriction in mice and invertebrates (Heintz et al. 2016; Lee et al. 2019a) and may also contribute to the superior stress resilience observed in NMRs (Lewis et al. 2012).

Superior stress response is a well-established characteristic of NMRs. NMRs have several enhanced stress resistance mechanisms including enhanced mitochondrial antioxidant defences, enhanced proteostasis and improved maintenance of genomic stability than do other shorter lived rodents (Perez et al. 2009; Petruseva et al. 2017; Munro et al. 2019). AS is another fundamental cellular stress resilience mechanism. The ability to adjust transcriptomic output has been associated with resistance to several endogenous and exogenous cellular stressors in plants and animals (Mastrangelo et al. 2012; Pai and Luca 2019). AS is governed by the combinatorial control of a series of splicing activators (SR proteins) and inhibitors (hnRNPs) (Smith and Valcarcel 2000). These regulators are dynamically expressed and responsive to multiple cellular stressors (Biamonti and Caceres 2009; Yamamoto et al. 2016; Kim Guisbert and Guisbert 2017; Jeffery et al. 2019). In humans and other species, splicing factor expression has been demonstrated to display altered expression in ageing and ageing phenotypes (Harries et al. 2011; Lee et al. 2016; Lee et al. 2019b; Lye et al. 2019). Conceptually, one might expect to see elevated expression of splicing enhancers and decreased expression of splicing silencers in species with conserved splicing regulation. However, each splice event is regulated by the combinatorial binding of a unique battery of splicing activators; inclusion of an exon therefore may rely upon the activation of one splice site, as well as the inhibition of a competitor site. For these reasons, we do not expect to see directionality changes at the level of the whole transcriptome. The data presented here suggest that not only do NMRs start with elevated splicing factor expression compared to similarly sized rodents, but their splicing regulatory capacity is well-maintained in old age.

Of all the organs, the brain is known for its diverse splicing patterns and requirement for correctly regulated splicing (Karlsson and Linnarsson 2017). This contributes to many aspects of brain function such as activity patterns and behavioural state (Que et al. 2019), correct configuration of neural circuits (Sudhof 2017) and neuronal differentiation, function and plasticity (Furlanis and Scheiffele 2018). Accordingly, dysregulation of splicing patterns is characteristic of many neurodegenerative and neurodevelopmental diseases in humans such as Alzheimer’s disease, Parkinson’s disease and Autism (Brudek et al. 2016; Parikshak et al. 2016; Raj et al. 2018). The reduced tolerance for splicing dysregulation in brain makes it an ideal organ to study the long term trajectory of splicing factor regulation. Dysregulated splicing factor and isoform expression is a feature of senescent astrocytes (Lye et al. 2019), as well as senescent skin and lung fibroblasts, endothelial cells and cardiomyocytes (Holly et al. 2013; Latorre et al. 2017; Latorre et al. 2018b). In other tissues, dysregulated splicing factor expression has also been suggested to be a driver of cellular senescence; targeted knockdown of the splicing activator *SRSF2* or the splicing inhibitor *HNRNPD* in early passage human primary endothelial cells is sufficient to induce senescence (Latorre et al. 2018c), and restoration of splicing factor expression using small molecule or genetic means in human primary skin and lung fibroblasts and endothelial cells rescues multiple aspects of the senescent cell phenotype (Latorre et al. 2017; Latorre et al. 2018a; Latorre et al. 2018c). The accumulation of senescent cells, whilst known to confer early life longevity benefits in terms of guarding against malignancy, contributing to wound repair and embryonic development (Attaallah et al. 2019) is known to be linked to ageing phenotypes. Targeted removal of such cells is able to bring about a remarkable rejuvenation of multiple aspects of age-related pathology in genetically modified animals (Baker et al. 2011; Baker et al. 2016; Baar et al. 2017; Baar et al. 2018; Baker and Petersen 2018). Newly emerging markers of senescence such as the chromatin remodelling component HMGB2 are elevated in senescent endothelial cells and demonstrably higher in the circulation of people on adverse ageing trajectories (Lawrence et al. 2018). These observations suggest that the accumulation of senescent cells may be at least partly causal to age-related phenotypes, and that new approaches such as Ramen microspectroscopy that are able to detect their accumulation in vivo (Liendl et al. 2019) may provide useful biomarkers to assess the efficacy of senotherapeutic interventions in the future.

The data presented here are consistent with a model by which the extreme longevity and conserved healthspan characteristic of NMRs may arise from conservation of alternative splicing regulation and maintenance of patterns of AS. This maintained transcriptomic plasticity could then ensure a more robust response to internal and external environmental stressors and avoidance of cellular senescence. These data add weight to the hypothesis that splicing regulation is a key feature in the avoidance of cellular senescence, and provide a potential explanation for the extreme longevity seen in NMRs. In the future, the genes and pathways responsible for maintenance of splicing regulation and molecular stress response may be promising future therapeutic targets for healthspan and lifespan interventions.

## Electronic supplementary material

ESM 1 (DOCX 637 kb)
