# Supplementary material for: Negligible senescence in naked mole rats may be a consequence of well-maintained splicing regulation
Source: GeroScience. 2020 Jan 11;42(2):633–51. doi: 10.1007/s11357-019-00150-7 (PMC7205774; doi:10.1007/s11357-019-00150-7)
Supplement: Supplementary file 1 — (DOCX 637 kb) [file 11357_2019_150_MOESM1_ESM.docx]

**Supplementary Figure S1: Isoform structures of cognition- and senescence-related genes measured in this study**

Shown here are gene structures and locations of custom-designed qPCR assays to measure isoform expression levels.

**
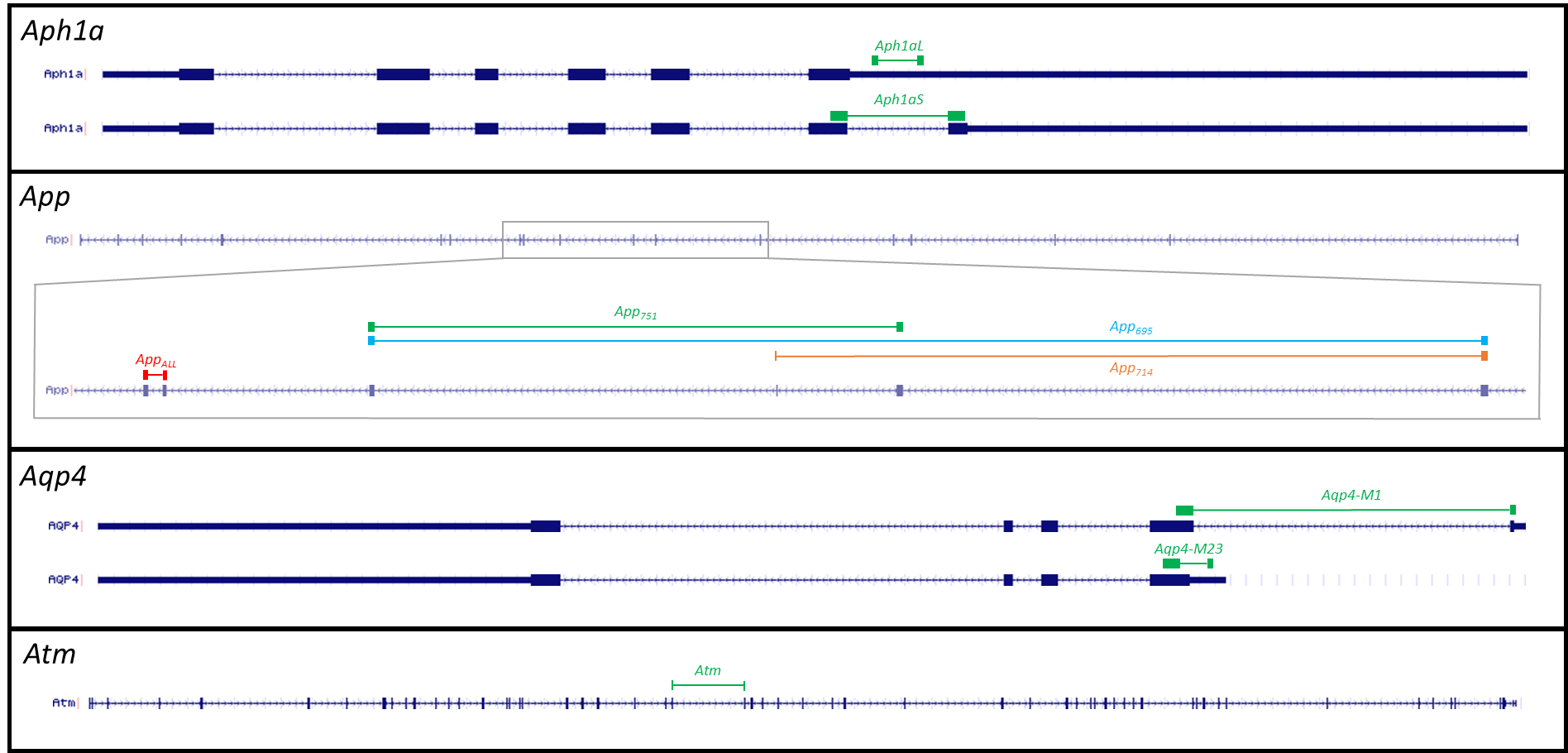
**

**Supplementary Figure S1: Continued**

**
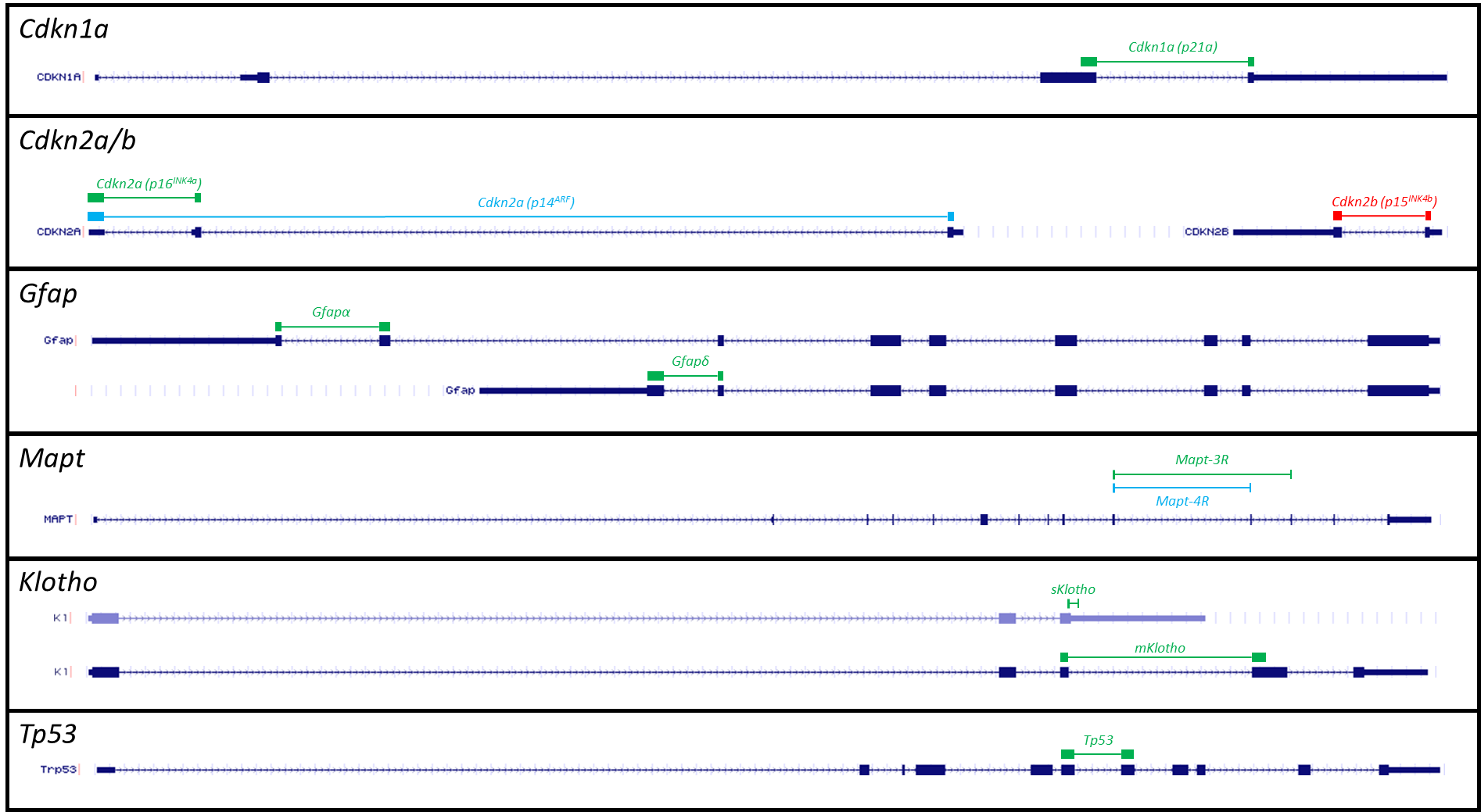
**

**Supplementary figure S2: Decline in splicing factor expression and disruption of patterns of alternative splicing in senescent human astrocytes.**

**a.** Splicing factor expression differences in early and late passage human astrocytes are given here. **b.** Changes in the expression of alternatively expressed isoforms of genes with known roles in brain function in early and late passage human astrocytes are given here. Data are adapted from Lye et al., 2019.


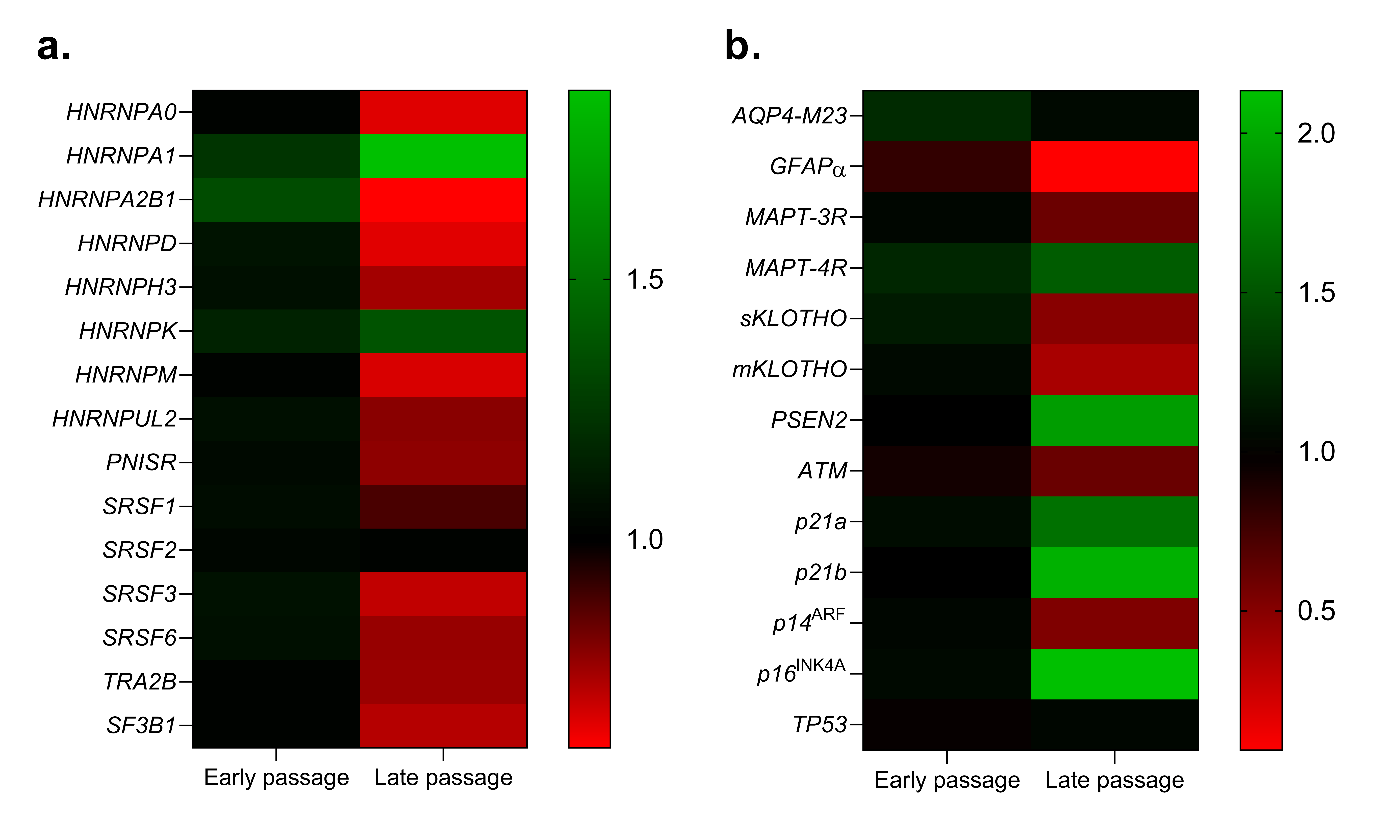


**Supplementary Table S1: Genome assemblies used for sequence alignment**

Details of assemblies used to obtain sequence data for alignment of genes of interest prior to assay design.

| **Species** | | **Genome assembly** | |
| --- | --- | --- | --- |
| Guinea pig | *(Cavia porcellus)* | Feb. 2008 | (Broad/cavpor3) |
| Human | *(Homo sapiens)* | Dec. 2013 | (GRCh38/hg38) |
| Mouse | *(Mus musculus)* | Dec. 2011 | (GRCm38/mm10) |
| Naked mole-rat | *(Heterocephalus glaber)* | Jan. 2012 | (Broad HetGla_female_1.0/hetgla2) |
| Rat | *(Rattus norvegicus)* | Jul. 2014 | (RGSC 6.0/rn6) |

**Supplementary Table S2: RT-PCR and sequencing primer sequences**

Details of primer sequences, PCR kits used and cycling conditions for all RT-PCR carried out. M13 sequencing tags are shown in bold within square brackets.

|  | **Gene name** | **Forward primer** | **Reverse primer** | **Included in analysis** | **PCR Kit used** | **Annealing Temp (°C)** | **No. of cycles** |
| --- | --- | --- | --- | --- | --- | --- | --- |
| Endogenous controls | *Idh3b* | **[TGTAAAACGACGGCCAGT]**AGGAGCACCACCTGAGTGA | **[CAGGAAACAGCTATGACC]**TTAGCCGCATATCATAGGAAGC | Y | Megamix Royal | 55 | 35 |
|  | *Polr2a* | **[TGTAAAACGACGGCCAGT]**TGGAGCTGTATGCTGAGTGG | **[CAGGAAACAGCTATGACC]**GAGCTGCTGCACCATTCTG | Y | Megamix Royal | 55 | 35 |
|  | *Ppia* | **[TGTAAAACGACGGCCAGT]**CTGTTTGCAGACAAAGTTCC | **[CAGGAAACAGCTATGACC]**TGACACATAAATCCTGGAATAATT | Y | Megamix Royal | 55 | 35 |
|  | *Tbp* | **[TGTAAAACGACGGCCAGT]**ACGGCGCTGATTTTCAGTT | **[CAGGAAACAGCTATGACC]**GCACGAGACCCTCCAATCTTATA | Y | Megamix Royal | 55 | 35 |
| Splicing factors | *Hnrnpa0* | **[TGTAAAACGACGGCCAGT]**TTCGAGGCCTTCGGGACT | **[CAGGAAACAGCTATGACC]**GGTGCCGAACTGCGAGAA | Y | Megamix Royal | 55 | 35 |
|  | *Hnrnpa1* | **[TGTAAAACGACGGCCAGT]**AACACCAAGCGCTCCAGG | **[CAGGAAACAGCTATGACC]**CCCCTCTTCTTCCCACTGC | Y | Megamix Royal | 55 | 35 |
|  | *Hnrnpa2b1* | **[TGTAAAACGACGGCCAGT]**CAACTTTGGTTTTGGGGATTC | **[CAGGAAACAGCTATGACC]**GTCCTCCTCCATACCCATTATAGC | Y | Megamix Royal | 55 | 35 |
|  | *Hnrnpd* | **[TGTAAAACGACGGCCAGT]**AATGGGAAGGTGATTGATCCTA | **[CAGGAAACAGCTATGACC]**CTAAGCCCAACATTGTGGTATTT | Y | Megamix Royal | 55 | 35 |
|  | *Hnrnph3* | **[TGTAAAACGACGGCCAGT]**ACGGTATTGAGATGGATTGGGTTA | **[CAGGAAACAGCTATGACC]**TGTGTTTCCCCAGAGCATTTT | Y | Megamix Royal | 55 | 35 |
|  | *Hnrnpk* | **[TGTAAAACGACGGCCAGT]**ATTCCACTGACCGAGTTGTTCTT | **[CAGGAAACAGCTATGACC]**AAATCCTACAGGCCGTCCA | Y | Megamix Royal | 55 | 35 |
|  | *Hnrnpm* | **[TGTAAAACGACGGCCAGT]**AATGCACTGAAGAGAGGAGAGATC | **[CAGGAAACAGCTATGACC]**ACCCCCAATGCGGTCAAT | Y | Megamix Royal | 55 | 35 |
|  | *Hnrnpul2* | **[TGTAAAACGACGGCCAGT]**GAAGCTCAGCCCATTGTCA | **[CAGGAAACAGCTATGACC]**CCCTCTGTTTCCAGACTGC | Y | Megamix Royal | 55 | 35 |
|  | *Pnisr* | **[TGTAAAACGACGGCCAGT]**GATGGAACAACAACGTTCACA | **[CAGGAAACAGCTATGACC]**CTTCTTCAGTCATCTCTGGTTCAC | Y | Megamix Royal | 55 | 35 |
|  | *Srsf1* | **[TGTAAAACGACGGCCAGT]**GAAGCGGCCGTGGTACAG | **[CAGGAAACAGCTATGACC]**CAAACTCCACGACACCAGTGC | Y | Megamix Royal | 55 | 35 |
|  | *Srsf2* | **[TGTAAAACGACGGCCAGT]**TCGCTACAGCCGCTCCAA | **[CAGGAAACAGCTATGACC]**ATGGACCGATGGACTGAGTTC | Y | Megamix Royal | 55 | 35 |
|  | *Srsf3* | **[TGTAAAACGACGGCCAGT]**GGAACGGGCTTTCGGCTATTA | **[CAGGAAACAGCTATGACC]**ATCTCGAGGGCGACGACC | Y | Megamix Royal | 55 | 35 |
|  | *Srsf6* | **[TGTAAAACGACGGCCAGT]**TTACGAGCTGAACGGCAAAG | **[CAGGAAACAGCTATGACC]**TTGCCAACTACAACGACTAGAAAG | Y | Megamix Royal | 55 | 35 |
|  | *Tra2b* | **[TGTAAAACGACGGCCAGT]**TTGATGGGCGTAGAATCAGAG | **[CAGGAAACAGCTATGACC]**TAGTAGTCCCGATCATCATAGCC | Y | Megamix Royal | 55 | 35 |
|  | *Sf1* | **[TGTAAAACGACGGCCAGT]**ACTCGCAGCATTACCAACACTAC | **[CAGGAAACAGCTATGACC]**CGGTGGAGGTGGATTATTGG | Y | Megamix Royal | 55 | 35 |
|  | *Sf3b1* | **[TGTAAAACGACGGCCAGT]**CGAGAGATTCAAGGCAAGAAGG | **[CAGGAAACAGCTATGACC]**TGTTCTGTTGACTGTGGGATATCA | Y | Megamix Royal | 55 | 35 |

**Supplementary Table S2: Continued**

|  | ***Gene name*** | **Forward primer** | **Reverse primer** | **Included in analysis** | **PCR Kit used** | **Annealing Temp (°C)** | **No. of cycles** |
| --- | --- | --- | --- | --- | --- | --- | --- |
| Alternate isoforms of cognition-related genes | *Aph1a* **(nested)** | **[TGTAAAACGACGGCCAGT]**GGGTCCCTCCGAAGTATCCA | **[CAGGAAACAGCTATGACC]**AGTCCTCGGGTGGGATGC | Y | Platinum II Taq | 55 | 40 |
|  | *App* **(nested)** | **[TGTAAAACGACGGCCAGT]**TGAAGAAGCCACAGAGAAAACC | **[CAGGAAACAGCTATGACC]**AGATACTTGTCAACGGCGTCA | Y | Platinum II Taq | Unsuccessful | |
|  | *Aqp4-M1* | **[TGTAAAACGACGGCCAGT]**CCAAAACCCAATCAGACGA | **[CAGGAAACAGCTATGACC]**GAAATTCTGCGGTGACTGC | Y | Platinum II Taq | 55 | 35 |
|  | *Aqp4-M23* | **[TGTAAAACGACGGCCAGT]**CCTTGCCCCTAACTCCATAAA | **[CAGGAAACAGCTATGACC]**AGCAGGACAAAGATAAGCATAGCC | Y | Platinum II Taq | 58 | 40 |
|  | *Gfapα* | **[TGTAAAACGACGGCCAGT]**TCAAGCTGGCCCTGGACAT | **[CAGGAAACAGCTATGACC]**CGCAGGCCTAGTGTGACAGAG | Y | Platinum II Taq | 55 | 35 |
|  | *Gfapδ* | **[TGTAAAACGACGGCCAGT]**ATGGAGATTGCCACCTACCG | **[CAGGAAACAGCTATGACC]**CATGGTGAGGCTTTTGAGGTAT | Y | Platinum II Taq | 53 | 40 |
|  | *mKlotho* | **[TGTAAAACGACGGCCAGT]**GGATGGGGTTGATGTCATTG | **[CAGGAAACAGCTATGACC]**GAGAAGTGAAAATGCGTGACGT | Y | Platinum II Taq | 58 | 40 |
|  | *sKlotho* | **[TGTAAAACGACGGCCAGT]**GACGTGGACTCTTCTATGTCGA | **[CAGGAAACAGCTATGACC]**GATGGCTTTAAGAGGGATTGAAAA | Y | Platinum II Taq | 55 | 40 |
|  | *Mapt* **(nested)** | **[TGTAAAACGACGGCCAGT]**AAGGTAGCAGTGGTCCGCACTC | **[CAGGAAACAGCTATGACC]**TGCTCAGATCCACTGGTTTGTAGA | Y | Platinum II Taq | 55 | 40 |
|  | *Psen1 (ins_TAC_)* | **[TGTAAAACGACGGCCAGT]**GCGCCAAACATGTCATCA | **[CAGGAAACAGCTATGACC]**GGCATGGATGACCTTATAGCAC | **N** | Platinum II Taq | 58 | 40 |
|  | *Psen1 (VRSQ)* | **[TGTAAAACGACGGCCAGT]**ACAGTTGCTCCAATGACAGAGTT | **[CAGGAAACAGCTATGACC]**ACCACCTGCCGTGAGTTCC | **N** | Platinum II Taq | 55 | 35 |
|  | *Psen2* **(nested)** | **[TGTAAAACGACGGCCAGT]**CAAGTCCGTGCGCTTCTACA | **[CAGGAAACAGCTATGACC]**AAGAGCGTGGGGTAGTCCAT | **N** | Platinum II Taq | 55 | 40 |
| Senescence-related genes | *Atm* | **[TGTAAAACGACGGCCAGT]**CAAGCAGCTGAGACAAATAATGTT | **[CAGGAAACAGCTATGACC]**AGGGGTATAAGTGTGCCAACAAT | Y | Platinum II Taq | 58 | 40 |
|  | *Cdkn1a (p21a)* | **[TGTAAAACGACGGCCAGT]**GGGGACGCCGAGACACAC | **[CAGGAAACAGCTATGACC]**GTGGGCTCCCCTGTTTCTG | Y | Platinum II Taq | 58 | 40 |
|  | *Cdkn2a (p14^ARF^)* | **[TGTAAAACGACGGCCAGT]**CACGGCAGGCAGGAGAGT | **[CAGGAAACAGCTATGACC]**AGCGCCACCAGAGTATCCA | Y | Platinum II Taq | 55 | 35 |
|  | *Cdkn2a (p16^INK4a^)* | **[TGTAAAACGACGGCCAGT]**CCAGGAGGTACGCGAGCT | **[CAGGAAACAGCTATGACC]**CGTCATGCACCGGTAGTGT | Y | Platinum II Taq | 56 | 35 |
|  | *Cdkn2b (p15^INK4b^)* | **[TGTAAAACGACGGCCAGT]**AGGGCAAGTGGAGACGGT | **[CAGGAAACAGCTATGACC]**GTGAGTGTGGCGGGGTCT | Y | Platinum II Taq | 55 | 35 |
|  | *Tp53* | **[TGTAAAACGACGGCCAGT]**GCGCTGCTCCGATAGTGATG | **[CAGGAAACAGCTATGACC]**GATGGGCCTACGGTTCATGC | Y | Platinum II Taq | 52 | 40 |
| Band-stab primers | *Aph1a* | TGGTTGGGAGTCACCTACTGACA | GCAACCTGCACTGTCCAGAAC | Y | Platinum II Taq | 55 | 40 |
|  | *App* | CCACCACCACGGAGTCTGT | GTGCTGGCTGCTGTGGTA | Y | Platinum II Taq | Unsuccessful | |
|  | *Mapt* | ATCGCAGTGGCTACAGCA | CCACCTCCTGGTTTATGATGA | Y | Platinum II Taq | 55 | 40 |
|  | *Psen2* | TGCTGTTTGTGCCCGTCA | GCCCTTCCAGTGGATGCA | **N** | Platinum II Taq | 55 | 40 |

**Supplementary Table S3: Taqman® assays used in this study**

Details of primer and reporter sequences, assay efficiencies and linearity (r^2^) of all custom Taqman® assays used in this study.

|  | **Assay Name** | **Forward Primer Sequence** | **Reverse Primer Sequence** | **Reporter Sequence** | **Assay efficiency (%)** | **Standard curve r^2^** |
| --- | --- | --- | --- | --- | --- | --- |
| Endogenous controls | *Idh3b* | TGCTGAATTCCATGAAGGAGAACAA | GCCGCATATCATAGGAAGCTAGTTC | CCATCATTGGAAAGATCC | 97.445 | 0.998 |
|  | *Polr2a* | CGGCCTGCTGTGGTGAT | CCGCAACTGATTATTGATCTTCACAA | CAAGTCATCCTGGTTTCG | 92.665 | 0.999 |
|  | *Ppia* | CTGTTTGCAGACAAAGTTCCAAAG | TCCTTTCTCTCCAGTGCTCAGA | ACAGCAGAAAACTTTC | 99.536 | 0.999 |
|  | *Tbp* | CCACGGCGCTGATTTTCA | TTGCAGCTAACCTGGACTGTT | CTTCACTCTTGGCTCCC | 99.149 | 0.997 |
| Splicing factors | *Hnrnpa0* | CCGACGCCGCCATG | GCCGCGCCGAATCC | CAGCTCCACGGTGTTAC | 101.021 | 0.993 |
|  | *Hnrnpa1* | GGAAGAGTTGTGGAACCAAAGAGA | GGGCACCCGGTCTTTGA | CTGTCTCAAGAGAAGATTC | 99.586 | 0.999 |
|  | *Hnrnpa2b1* | CCAGGACCAGGAAGTAACTTTAGAG | ATCACCAAATCCACGTCCACTT | CATATCCGTCAGATCCTC | 99.058 | 1.000 |
|  | *Hnrnpd* | CACCTGAAGAGAAAATAAGGGAGTACTT | AGAATCCACGCCTCTTATTGGTTTT | TCCACCTCACCAAAACC | 97.216 | 0.998 |
|  | *Hnrnph3* | GTTGCAGCAAAGAGGAAATAGTTCA | CCTGGTAGTCCATCGTCAATGTTAT | CCAACCCTTGAAAGAAC | 94.290 | 0.999 |
|  | *Hnrnpk* | GGTTGTAGAGTGCATAAAGATCATCCT | GATCATAAGGTTGCGCACGTC | TCTGAGTCTCCCATCAAAG | 92.773 | 0.999 |
|  | *Hnrnpm* | GAGAGGAGAGATCATTGCAAAGCA | GGGCCCATCCTCTCAATCC | ACCTCCACCTCCTCCC | 98.878 | 0.997 |
|  | *Hnrnpul2* | GCGCAACCGGCAGAAC | GGCCCGGCTGTCGTA | CTGCCCGCCCACGTAG | 97.676 | 0.996 |
|  | *Pnisr* | GCCCTCGTTTACCTCAGAGAA | GCTTGCAGCCTCAATGTTTTCAG | CTTCATCACTATCAAATTTAC | 92.022 | 0.999 |
|  | *Srsf1* | CCGGCGGTCTGAAAACAG | AAATCCTGCCAGCTTCCACTT | TTGTCTCTGGACTTCCTCC | 97.760 | 0.999 |
|  | *Srsf2* | CCAAGTCTCCAGAAGAGGAAGGA | CCCCAAAGTCCTCTGTTATGCT | TTGCCGATATATCATTTTCT | 93.281 | 0.998 |
|  | *Srsf3* | CCGAGATGCAGCTGATGCT | CATTGGACAGTTCCACTCTGACT | CTAGATGGAAGAACATTATGC | 97.533 | 0.999 |
|  | *Srsf6* | CGCGACGGCTACAGCTA | GTTCTCCGACTGCTGTATCCA | CACCACTGCGGCTTC | 98.508 | 0.998 |
|  | *Tra2b* | ACCCCAACACCAGGAATTTACATG | AGCCACGGTCATATCCTCTGT | CCTACCTATGGCAGTTCAC | 97.573 | 0.999 |
|  | *Sf1* | GGCCACATTGCTTCAGATTGC | CGTGCTTTATCCTGAGCTGACT | CAGGCCTCTGGAACTT | 97.390 | 0.998 |
|  | *Sf3b1* | GCTGGCTATGTCACATCAATTGC | CCAAGCAAGCTCGTCGATGAT | TCGTCATCATCTTCAAGTTC | 94.882 | 0.999 |

**Supplementary Table S3: Continued**

|  | ***Assay Name*** | **Forward Primer Sequence** | **Reverse Primer Sequence** | **Reporter Sequence** | **Assay efficiency (%)** | **Standard curve r2** |
| --- | --- | --- | --- | --- | --- | --- |
| Alternate isoforms of cognition-related genes | *Aph1aL* | GGGTCCCTCCGAAGTATCCA | GAATACACCATCACCCGACTGT | CAGCCTCTCGTGCCGAC | 87.832 | 0.998 |
|  | *Aph1aS* | ACCTTCAGGGTTTTGCATTGC | GATTCAAACCCACCAGTCACTGA | CACCAAAGGTCACAAATG | 99.026 | 0.998 |
|  | *App_695_* | CACCACCACGGAGTCTGT | GGCGTCTCGAGATACTTGTCAAC | CTGTGGTAGGAACTCGAACC | 98.695 | 0.999 |
|  | *App_714_* | CACCACCACGGAGTCTGT | GGTTCCCCGGTAGTCTTGAGTA | TCGAGTGTCCCAAAGTC | 81.417 | 0.934 |
|  | *App_751_* | ACTGCATGGCGGTGTGT | GGCGTCTCGAGATACTTGTCAAC | CTGTGGTAGGAATGACG | 101.132 | 0.999 |
|  | *App_ALL_* | ACTTGCCCAAGGCTGATAAGAAG | CTCCAGGGACTCCACTTTCTC | CTGTGATTCAGCATTTCC | 99.939 | 0.999 |
|  | *Aqp4-M1* | CAGGGAAGGCATGAGTGATAGAC | CCATGATGTTCTCTCTGGTACACAA | ACACTTGCCCCACCGCC | 97.964 | 0.998 |
|  | *Aqp4-M23* | TTGCAATTTTGAGGCTGATTTGGT | CCATGATGTTCTCTCTGGTACACAA | TCCACACTTGCTGAAAAG | 88.802 | 0.992 |
|  | *Gfapα* | CGTGCTCAAGACAGTGGAGATG | CCTTGTGCTCCTGCTTGGA | CCTGAGTGACCTCCCC | 92.802 | 0.999 |
|  | *Gfapδ* | CGTGCAGACTTTCTCCAACCT | GCGGACCTCCCCTTTTGT | TTGCCTCCTCGGATCTG | 99.400 | 0.988 |
|  | *Mapt-3R* | GGCTCTACCGAGAACCTGAAG | GAAGTCACCTTGCTCAGATCCA | TTGTAGACTATTTGCACCTTCC | 101.457 | 0.994 |
|  | *Mapt-4R* | GGCTCTACCGAGAACCTGAAG | GACGTTGCTAAGATCCAGCTTCT | CTGCACCTTCCCACCTCC | 97.149 | 0.997 |
|  | *mKlotho* | GACATTTCCCTGTGACTTTGCTT | CGTTGGTGTCGGTAAACTGAGA | CAGAGTGGTGTCTACTTGAA | 91.566 | 0.989 |
|  | *sKlotho* | GACATTTCCCTGTGACTTTGCTT | CACTGCTGGTTGGTTTTGTCA | CAACCACATTCAAGTAAGTCA | 100.298 | 0.991 |
| Senescence-related genes | *Atm* | GGGCCTTTGTTCTTCGAGATGTTAT | GCTACGTAATGACACATCCATGAGA | ATTAACCAAAGGACTTCCC | 86.671 | 0.999 |
|  | *Cdkn1a (p21a)* | ACCTCTCAGAGCCGAAAACG | AGGCGGCGCTTGGA | CAGCATGACGGATTTCTA | 96.838 | 0.985 |
|  | *Cdkn2a (p14^ARF^)* | GCAGCAGCCGTATCCTAGAAG | CAGCGCGGCCACTTG | TTGCCCATCATCATGACCTGG | 88.855 | 0.943 |
|  | *Cdkn2a (p16^INK4a^)* | GCCCGGAACCGTTTCG | CGGCCACTTGGGTGTTG | CATCATCATGACCTGAATCG | 94.577 | 0.992 |
|  | *Cdkn2b (p15^INK4b^)* | CCCAACGCCGTCAATCG | GCAGAAGCTCAGCCATGTG | CCCGATCCAGGTCATG | 93.150 | 0.963 |
|  | *Tp53* | CGTGGTGGTACCCTATGATCTG | CACATATAGTTGTAGTGGATGGTGGTA | CAGTCAGAGCCAACCTCA | 98.383 | 0.994 |

**Supplementary Table S4: Species comparison data**

Comparison of splicing factor expression levels between naked mole-rat and mouse in two different tissues, by t-test. Transcripts which meet FDR correction for multiple testing (p<0.0381) are shown in bold italic and underlined. SE: standard error, 95% CI: 95% confidence intervals.

| **Brain** | | | | | | **Spleen** | | | | | |
| --- | --- | --- | --- | --- | --- | --- | --- | --- | --- | --- | --- |
|  | Mean Difference | SE | p-value | 95% CI upper | 95% CI lower |  | Mean Difference | SE | p-value | 95% CI upper | 95% CI lower |
| *Hnrnpa0* | -0.93 | 0.18 | ***<0.001*** | -0.55 | -1.31 | *Hnrnpa0* | -0.74 | 0.06 | ***<0.001*** | -0.62 | -0.87 |
| *Hnrnpa1* | 0.00 | 0.05 | 0.978 | 0.12 | -0.11 | *Hnrnpa1* | 0.67 | 0.03 | ***<0.001*** | 0.73 | 0.61 |
| *Hnrnpa2b1* | -0.01 | 0.06 | 0.883 | 0.12 | -0.14 | *Hnrnpa2b1* | 0.26 | 0.03 | ***<0.001*** | 0.32 | 0.20 |
| *Hnrnpd* | 0.17 | 0.05 | ***0.005*** | 0.28 | 0.06 | *Hnrnpd* | 0.19 | 0.03 | ***<0.001*** | 0.26 | 0.12 |
| *Hnrnph3* | 0.50 | 0.04 | ***<0.001*** | 0.58 | 0.41 | *Hnrnph3* | 0.59 | 0.02 | ***<0.001*** | 0.64 | 0.54 |
| *Hnrnpk* | 0.45 | 0.05 | ***<0.001*** | 0.55 | 0.34 | *Hnrnpk* | 0.38 | 0.03 | ***<0.001*** | 0.44 | 0.32 |
| *Hnrnpm* | -0.07 | 0.09 | 0.453 | 0.13 | -0.28 | *Hnrnpm* | -0.10 | 0.03 | ***0.013*** | -0.02 | -0.17 |
| *Hnrnpul2* | -1.05 | 0.12 | ***<0.001*** | -0.77 | -1.32 | *Hnrnpul2* | -0.42 | 0.04 | ***<0.001*** | -0.33 | -0.51 |
| *Pnisr* | 0.47 | 0.10 | ***<0.001*** | 0.68 | 0.26 | *Pnisr* | 0.26 | 0.02 | ***<0.001*** | 0.30 | 0.21 |
| *Srsf1* | 0.51 | 0.07 | ***<0.001*** | 0.67 | 0.35 | *Srsf1* | 0.46 | 0.04 | ***<0.001*** | 0.54 | 0.38 |
| *Srsf2* | 0.67 | 0.08 | ***<0.001*** | 0.85 | 0.49 | *Srsf2* | 0.49 | 0.03 | ***<0.001*** | 0.56 | 0.42 |
| *Srsf3* | 1.45 | 0.05 | ***<0.001*** | 1.56 | 1.34 | *Srsf3* | 1.58 | 0.03 | ***<0.001*** | 1.64 | 1.51 |
| *Srsf6* | 0.74 | 0.09 | ***<0.001*** | 0.94 | 0.55 | *Srsf6* | 0.64 | 0.04 | ***<0.001*** | 0.73 | 0.55 |
| *Tra2b* | 0.26 | 0.06 | ***0.001*** | 0.38 | 0.13 | *Tra2b* | 0.35 | 0.05 | ***<0.001*** | 0.46 | 0.23 |
| *Sf1* | 0.32 | 0.14 | ***0.042*** | 0.62 | 0.01 | *Sf1* | 0.29 | 0.03 | ***<0.001*** | 0.36 | 0.23 |
| *Sf3b1* | 0.08 | 0.07 | 0.241 | 0.23 | -0.06 | *Sf3b1* | -0.06 | 0.01 | ***0.001*** | -0.03 | -0.09 |

**Supplementary Table S5: Splicing factor expression levels in naked mole-rat brain across a 22-year time series**

Splicing factor expression levels in brain tissue of naked mole-rats from fetus through to 22-year-old, stratified into age groups, by linear regression. Transcripts showing nominal associations (p<0.05) are shown in italic and underlined, those which meet FDR correction for multiple testing (p<0.00158) are shown in bold italic and underlined. SE: standard error, 95% CI: 95% confidence intervals.

| ***Hnrnpa0*** | | | | | |  |  | ***Hnrnpa1*** | | | | | |
| --- | --- | --- | --- | --- | --- | --- | --- | --- | --- | --- | --- | --- | --- |
|  | β-coefficient | SE | *p*-value | 95% CI upper | 95% CI lower |  |  |  | β-coefficient | SE | *p*-value | 95% CI upper | 95% CI lower |
| Fetus | 0.741 | 0.224 | *0.002* | 1.193 | 0.289 |  |  | Fetus | 2.264 | 0.114 | ***<0.001*** | 2.494 | 2.035 |
| 1 Day | -0.053 | 0.204 | 0.796 | 0.359 | -0.466 |  |  | 1 Day | 1.022 | 0.104 | ***<0.001*** | 1.231 | 0.813 |
| 2 Weeks | -0.624 | 0.250 | *0.017* | -0.119 | -1.129 |  |  | 2 Weeks | 0.483 | 0.127 | ***<0.001*** | 0.739 | 0.227 |
| 3-4 Years | 0 | 0.177 | 1 | 0.357 | -0.357 |  |  | 3-4 Years | 0 | 0.090 | 1 | 0.181 | -0.181 |
| 5-6 Years | -0.359 | 0.250 | 0.158 | 0.146 | -0.865 |  |  | 5-6 Years | 0.086 | 0.127 | 0.502 | 0.342 | -0.170 |
| 7-8 Years | -0.451 | 0.250 | 0.079 | 0.054 | -0.956 |  |  | 7-8 Years | 0.076 | 0.127 | 0.553 | 0.332 | -0.180 |
| 9-12 Years | 0.069 | 0.204 | 0.737 | 0.482 | -0.344 |  |  | 9-12 Years | -0.184 | 0.104 | 0.083 | 0.025 | -0.394 |
| 13-15 Years | -0.366 | 0.224 | 0.110 | 0.086 | -0.817 |  |  | 13-15 Years | 0.041 | 0.114 | 0.721 | 0.270 | -0.188 |
| 17-20 Years | -0.221 | 0.224 | 0.329 | 0.231 | -0.673 |  |  | 17-20 Years | 0.165 | 0.114 | 0.153 | 0.395 | -0.064 |
| 21-22 Years | -0.385 | 0.250 | 0.132 | 0.121 | -0.890 |  |  | 21-22 Years | 0.164 | 0.127 | 0.204 | 0.420 | -0.093 |
| ***Hnrnpa2b1*** | | | | | |  |  | ***Hnrnpd*** | | | | | |
|  | β-coefficient | SE | *p*-value | 95% CI upper | 95% CI lower |  |  |  | β-coefficient | SE | *p*-value | 95% CI upper | 95% CI lower |
| Fetus | 0.997 | 0.123 | ***<0.001*** | 1.245 | 0.749 |  |  | Fetus | -0.056 | 0.064 | 0.382 | 0.073 | -0.185 |
| 1 Day | 0.457 | 0.112 | ***<0.001*** | 0.683 | 0.230 |  |  | 1 Day | -0.204 | 0.058 | ***0.001*** | -0.086 | -0.322 |
| 2 Weeks | 0.276 | 0.137 | 0.051 | 0.553 | -0.001 |  |  | 2 Weeks | -0.370 | 0.071 | ***<0.001*** | -0.225 | -0.514 |
| 3-4 Years | 0 | 0.097 | 1 | 0.196 | -0.196 |  |  | 3-4 Years | 0 | 0.051 | 1 | 0.102 | -0.102 |
| 5-6 Years | 0.106 | 0.137 | 0.443 | 0.384 | -0.171 |  |  | 5-6 Years | -0.049 | 0.071 | 0.493 | 0.095 | -0.194 |
| 7-8 Years | 0.156 | 0.137 | 0.263 | 0.433 | -0.121 |  |  | 7-8 Years | 0.064 | 0.071 | 0.375 | 0.208 | -0.080 |
| 9-12 Years | -0.079 | 0.112 | 0.485 | 0.148 | -0.306 |  |  | 9-12 Years | 0.047 | 0.058 | 0.422 | 0.165 | -0.071 |
| 13-15 Years | -0.009 | 0.123 | 0.941 | 0.239 | -0.257 |  |  | 13-15 Years | -0.072 | 0.064 | 0.263 | 0.057 | -0.201 |
| 17-20 Years | 0.055 | 0.123 | 0.658 | 0.303 | -0.193 |  |  | 17-20 Years | -0.025 | 0.064 | 0.699 | 0.104 | -0.154 |
| 21-22 Years | 0.162 | 0.137 | 0.245 | 0.440 | -0.115 |  |  | 21-22 Years | -0.093 | 0.071 | 0.200 | 0.051 | -0.237 |
| ***Hnrnph3*** | | | | | |  |  | ***Hnrnpk*** | | | | | |
|  | β-coefficient | SE | *p*-value | 95% CI upper | 95% CI lower |  |  |  | β-coefficient | SE | *p*-value | 95% CI upper | 95% CI lower |
| Fetus | 0.743 | 0.091 | ***<0.001*** | 0.927 | 0.559 |  |  | Fetus | 0.664 | 0.090 | ***<0.001*** | 0.846 | 0.481 |
| 1 Day | -0.036 | 0.083 | 0.663 | 0.131 | -0.204 |  |  | 1 Day | 0.147 | 0.083 | 0.081 | 0.314 | -0.019 |
| 2 Weeks | -0.134 | 0.102 | 0.195 | 0.072 | -0.340 |  |  | 2 Weeks | -0.045 | 0.101 | 0.656 | 0.159 | -0.249 |
| 3-4 Years | 0 | 0.072 | 1 | 0.145 | -0.145 |  |  | 3-4 Years | 0 | 0.071 | 1 | 0.144 | -0.144 |
| 5-6 Years | 0.115 | 0.102 | 0.267 | 0.320 | -0.091 |  |  | 5-6 Years | 0.031 | 0.101 | 0.759 | 0.235 | -0.173 |
| 7-8 Years | 0.072 | 0.102 | 0.486 | 0.277 | -0.134 |  |  | 7-8 Years | 0.143 | 0.101 | 0.164 | 0.347 | -0.061 |
| 9-12 Years | -0.181 | 0.083 | *0.036* | -0.013 | -0.349 |  |  | 9-12 Years | -0.045 | 0.083 | 0.585 | 0.121 | -0.212 |
| 13-15 Years | 0.091 | 0.091 | 0.322 | 0.275 | -0.093 |  |  | 13-15 Years | -0.053 | 0.090 | 0.558 | 0.129 | -0.236 |
| 17-20 Years | 0.063 | 0.091 | 0.492 | 0.247 | -0.121 |  |  | 17-20 Years | -0.051 | 0.090 | 0.577 | 0.132 | -0.233 |
| 21-22 Years | 0.183 | 0.102 | 0.080 | 0.389 | -0.023 |  |  | 21-22 Years | 0.017 | 0.101 | 0.867 | 0.221 | -0.187 |
| ***Hnrnpm*** | | | | | |  |  | ***Hnrnpul2*** | | | | | |
|  | β-coefficient | SE | *p*-value | 95% CI upper | 95% CI lower |  |  |  | β-coefficient | SE | *p*-value | 95% CI upper | 95% CI lower |
| Fetus | -0.009 | 0.123 | 0.942 | 0.240 | -0.258 |  |  | Fetus | -0.898 | 0.260 | ***0.001*** | -0.372 | -1.424 |
| 1 Day | -0.262 | 0.113 | *0.025* | -0.035 | -0.490 |  |  | 1 Day | -1.191 | 0.238 | ***<0.001*** | -0.711 | -1.671 |
| 2 Weeks | -0.401 | 0.138 | *0.006* | -0.123 | -0.680 |  |  | 2 Weeks | -1.026 | 0.291 | ***0.001*** | -0.438 | -1.614 |
| 3-4 Years | 0 | 0.097 | 1 | 0.197 | -0.197 |  |  | 3-4 Years | 0 | 0.206 | 1 | 0.416 | -0.416 |
| 5-6 Years | -0.329 | 0.138 | *0.022* | -0.050 | -0.607 |  |  | 5-6 Years | -0.211 | 0.291 | 0.472 | 0.377 | -0.799 |
| 7-8 Years | -0.428 | 0.138 | *0.003* | -0.149 | -0.706 |  |  | 7-8 Years | -0.439 | 0.291 | 0.139 | 0.148 | -1.027 |
| 9-12 Years | -0.245 | 0.113 | *0.036* | -0.017 | -0.472 |  |  | 9-12 Years | -0.052 | 0.238 | 0.827 | 0.428 | -0.532 |
| 13-15 Years | -0.225 | 0.123 | 0.075 | 0.024 | -0.474 |  |  | 13-15 Years | 0.068 | 0.260 | 0.795 | 0.594 | -0.458 |
| 17-20 Years | 0.019 | 0.123 | 0.880 | 0.268 | -0.230 |  |  | 17-20 Years | -0.014 | 0.260 | 0.956 | 0.511 | -0.540 |
| 21-22 Years | -0.067 | 0.138 | 0.627 | 0.211 | -0.346 |  |  | 21-22 Years | -0.197 | 0.291 | 0.503 | 0.391 | -0.785 |

**Supplementary Table S5: (Continued)**

| ***Pnisr*** | | | | | |  |  | ***Srsf1*** | | | | | |
| --- | --- | --- | --- | --- | --- | --- | --- | --- | --- | --- | --- | --- | --- |
|  | β-coefficient | SE | *p*-value | 95% CI upper | 95% CI lower |  |  |  | β-coefficient | SE | *p*-value | 95% CI upper | 95% CI lower |
| Fetus | 0.610 | 0.162 | ***<0.001*** | 0.938 | 0.283 |  |  | Fetus | 1.050 | 0.118 | ***<0.001*** | 1.288 | 0.813 |
| 1 Day | 0.371 | 0.148 | *0.016* | 0.670 | 0.072 |  |  | 1 Day | 0.398 | 0.107 | ***<0.001*** | 0.615 | 0.181 |
| 2 Weeks | 0.403 | 0.181 | *0.032* | 0.769 | 0.036 |  |  | 2 Weeks | 0.219 | 0.131 | 0.104 | 0.484 | -0.047 |
| 3-4 Years | 0 | 0.128 | 1 | 0.259 | -0.259 |  |  | 3-4 Years | 0 | 0.093 | 1 | 0.188 | -0.188 |
| 5-6 Years | 0.117 | 0.181 | 0.523 | 0.483 | -0.249 |  |  | 5-6 Years | 0.024 | 0.131 | 0.856 | 0.289 | -0.242 |
| 7-8 Years | -0.150 | 0.181 | 0.414 | 0.217 | -0.516 |  |  | 7-8 Years | -0.108 | 0.131 | 0.415 | 0.157 | -0.374 |
| 9-12 Years | -0.270 | 0.148 | 0.075 | 0.029 | -0.569 |  |  | 9-12 Years | -0.156 | 0.107 | 0.154 | 0.061 | -0.373 |
| 13-15 Years | -0.116 | 0.162 | 0.478 | 0.211 | -0.444 |  |  | 13-15 Years | -0.128 | 0.118 | 0.284 | 0.110 | -0.365 |
| 17-20 Years | 0.217 | 0.162 | 0.188 | 0.544 | -0.111 |  |  | 17-20 Years | 0.154 | 0.118 | 0.198 | 0.391 | -0.084 |
| 21-22 Years | 0.273 | 0.181 | 0.140 | 0.639 | -0.094 |  |  | 21-22 Years | 0.176 | 0.131 | 0.188 | 0.441 | -0.090 |
| ***Srsf2*** | | | | | |  |  | ***Srsf3*** | | | | | |
|  | β-coefficient | SE | *p*-value | 95% CI upper | 95% CI lower |  |  |  | β-coefficient | SE | *p*-value | 95% CI upper | 95% CI lower |
| Fetus | 0.812 | 0.108 | ***<0.001*** | 1.029 | 0.594 |  |  | Fetus | 1.541 | 0.131 | ***<0.001*** | 1.804 | 1.277 |
| 1 Day | 0.149 | 0.098 | 0.136 | 0.348 | -0.049 |  |  | 1 Day | 0.663 | 0.119 | ***<0.001*** | 0.904 | 0.422 |
| 2 Weeks | -0.340 | 0.120 | *0.007* | -0.098 | -0.583 |  |  | 2 Weeks | 0.161 | 0.146 | 0.277 | 0.456 | -0.134 |
| 3-4 Years | 0 | 0.085 | 1 | 0.172 | -0.172 |  |  | 3-4 Years | 0 | 0.103 | 1 | 0.209 | -0.209 |
| 5-6 Years | 0.146 | 0.120 | 0.233 | 0.388 | -0.097 |  |  | 5-6 Years | 0.233 | 0.169 | 0.174 | 0.574 | -0.108 |
| 7-8 Years | 0.226 | 0.120 | 0.067 | 0.469 | -0.017 |  |  | 7-8 Years | 0.107 | 0.146 | 0.467 | 0.402 | -0.188 |
| 9-12 Years | 0.058 | 0.098 | 0.561 | 0.256 | -0.141 |  |  | 9-12 Years | -0.227 | 0.119 | 0.064 | 0.014 | -0.468 |
| 13-15 Years | -0.030 | 0.108 | 0.783 | 0.187 | -0.247 |  |  | 13-15 Years | 0.019 | 0.131 | 0.882 | 0.283 | -0.244 |
| 17-20 Years | -0.196 | 0.108 | 0.076 | 0.022 | -0.413 |  |  | 17-20 Years | 0.066 | 0.131 | 0.616 | 0.330 | -0.198 |
| 21-22 Years | 0.068 | 0.120 | 0.572 | 0.311 | -0.174 |  |  | 21-22 Years | 0.196 | 0.146 | 0.187 | 0.491 | -0.099 |
| ***Srsf6*** | | | | | |  |  | ***Tra2b*** | | | | | |
|  | β-coefficient | SE | *p*-value | 95% CI upper | 95% CI lower |  |  |  | β-coefficient | SE | *p*-value | 95% CI upper | 95% CI lower |
| Fetus | 1.786 | 0.144 | ***<0.001*** | 2.076 | 1.495 |  |  | Fetus | 1.257 | 0.113 | ***<0.001*** | 1.484 | 1.030 |
| 1 Day | 0.680 | 0.132 | ***<0.001*** | 0.946 | 0.415 |  |  | 1 Day | 0.406 | 0.103 | ***<0.001*** | 0.614 | 0.199 |
| 2 Weeks | 0.209 | 0.161 | 0.203 | 0.534 | -0.117 |  |  | 2 Weeks | 0.144 | 0.126 | 0.258 | 0.398 | -0.110 |
| 3-4 Years | 0.000 | 0.114 | 1 | 0.230 | -0.230 |  |  | 3-4 Years | 0.000 | 0.089 | 1 | 0.180 | -0.180 |
| 5-6 Years | 0.251 | 0.161 | 0.127 | 0.576 | -0.075 |  |  | 5-6 Years | 0.160 | 0.126 | 0.210 | 0.414 | -0.094 |
| 7-8 Years | 0.529 | 0.161 | ***0.002*** | 0.855 | 0.204 |  |  | 7-8 Years | 0.196 | 0.126 | 0.128 | 0.450 | -0.058 |
| 9-12 Years | 0.063 | 0.132 | 0.636 | 0.328 | -0.203 |  |  | 9-12 Years | 0.038 | 0.103 | 0.717 | 0.245 | -0.170 |
| 13-15 Years | -0.026 | 0.144 | 0.856 | 0.265 | -0.317 |  |  | 13-15 Years | -0.023 | 0.113 | 0.839 | 0.204 | -0.250 |
| 17-20 Years | 0.023 | 0.144 | 0.875 | 0.314 | -0.268 |  |  | 17-20 Years | 0.037 | 0.113 | 0.745 | 0.264 | -0.190 |
| 21-22 Years | 0.235 | 0.161 | 0.152 | 0.560 | -0.090 |  |  | 21-22 Years | 0.146 | 0.126 | 0.254 | 0.400 | -0.109 |
| ***Sf1*** | | | | | |  |  | ***Sf3b1*** | | | | | |
|  | β-coefficient | SE | *p*-value | 95% CI upper | 95% CI lower |  |  |  | β-coefficient | SE | *p*-value | 95% CI upper | 95% CI lower |
| Fetus | 0.430 | 0.223 | 0.061 | 0.881 | -0.020 |  |  | Fetus | 0.145 | 0.096 | 0.138 | 0.339 | -0.048 |
| 1 Day | -0.294 | 0.204 | 0.156 | 0.117 | -0.706 |  |  | 1 Day | 0.068 | 0.087 | 0.438 | 0.245 | -0.108 |
| 2 Weeks | 0.097 | 0.250 | 0.701 | 0.601 | -0.407 |  |  | 2 Weeks | -0.056 | 0.107 | 0.605 | 0.161 | -0.272 |
| 3-4 Years | 0 | 0.176 | 1 | 0.356 | -0.356 |  |  | 3-4 Years | 0 | 0.076 | 1 | 0.153 | -0.153 |
| 5-6 Years | -0.185 | 0.250 | 0.462 | 0.319 | -0.689 |  |  | 5-6 Years | 0.018 | 0.107 | 0.867 | 0.234 | -0.198 |
| 7-8 Years | -0.492 | 0.250 | 0.055 | 0.012 | -0.996 |  |  | 7-8 Years | 0.147 | 0.107 | 0.176 | 0.364 | -0.069 |
| 9-12 Years | -0.304 | 0.204 | 0.143 | 0.107 | -0.716 |  |  | 9-12 Years | 0.152 | 0.087 | 0.089 | 0.329 | -0.024 |
| 13-15 Years | -0.085 | 0.223 | 0.707 | 0.366 | -0.535 |  |  | 13-15 Years | -0.275 | 0.096 | *0.006* | -0.081 | -0.469 |
| 17-20 Years | 0.252 | 0.223 | 0.265 | 0.703 | -0.198 |  |  | 17-20 Years | -0.052 | 0.096 | 0.589 | 0.141 | -0.246 |
| 21-22 Years | -0.063 | 0.250 | 0.802 | 0.441 | -0.567 |  |  | 21-22 Years | -0.122 | 0.107 | 0.263 | 0.095 | -0.338 |

**Supplementary Table S6: Cognition-related isoform expression levels in naked mole-rat brain across a 22-year time series**

Cognition-related isoform expression levels in brain tissue of naked mole-rats from fetus through to 22-year-old, stratified into age groups, by linear regression. Transcripts showing nominal associations (p<0.05) are shown in italic and underlined, those which meet FDR correction for multiple testing (p<0.00195) are shown in bold italic and underlined. SE: standard error, 95% CI: 95% confidence intervals.

| ***Aph1aL*** | | | | | |  |  | ***Aph1aS*** | | | | | |
| --- | --- | --- | --- | --- | --- | --- | --- | --- | --- | --- | --- | --- | --- |
|  | β-coefficient | SE | *p*-value | 95% CI upper | 95% CI lower |  |  |  | β-coefficient | SE | *p*-value | 95% CI upper | 95% CI lower |
| Fetus | 0.411 | 0.101 | ***<0.001*** | 0.616 | 0.207 |  |  | Fetus | 1.423 | 0.169 | ***<0.001*** | 1.764 | 1.082 |
| 1 Day | -0.348 | 0.093 | ***0.001*** | -0.161 | -0.535 |  |  | 1 Day | 0.111 | 0.154 | 0.474 | 0.423 | -0.200 |
| 2 Weeks | 0.062 | 0.113 | 0.586 | 0.291 | -0.167 |  |  | 2 Weeks | 0.297 | 0.189 | 0.124 | 0.678 | -0.085 |
| 3-4 Years | 0 | 0.080 | 1 | 0.162 | -0.162 |  |  | 3-4 Years | 0 | 0.134 | 1 | 0.270 | -0.270 |
| 5-6 Years | -0.153 | 0.113 | 0.186 | 0.076 | -0.382 |  |  | 5-6 Years | -0.314 | 0.189 | 0.105 | 0.068 | -0.695 |
| 7-8 Years | 0.001 | 0.113 | 0.996 | 0.230 | -0.228 |  |  | 7-8 Years | -0.485 | 0.189 | *0.014* | -0.104 | -0.867 |
| 9-12 Years | 0.028 | 0.093 | 0.764 | 0.215 | -0.159 |  |  | 9-12 Years | -0.368 | 0.154 | *0.022* | -0.056 | -0.679 |
| 13-15 Years | -0.075 | 0.101 | 0.467 | 0.130 | -0.279 |  |  | 13-15 Years | -0.201 | 0.169 | 0.242 | 0.141 | -0.542 |
| 17-20 Years | -0.099 | 0.101 | 0.337 | 0.106 | -0.303 |  |  | 17-20 Years | 0.056 | 0.169 | 0.741 | 0.397 | -0.285 |
| 21-22 Years | -0.097 | 0.113 | 0.398 | 0.132 | -0.326 |  |  | 21-22 Years | -0.235 | 0.189 | 0.220 | 0.146 | -0.617 |
| ***App_695_*** | | | | | |  |  | ***App_714_*** | | | | | |
|  | β-coefficient | SE | *p*-value | 95% CI upper | 95% CI lower |  |  |  | β-coefficient | SE | *p*-value | 95% CI upper | 95% CI lower |
| Fetus | -0.809 | 0.145 | ***<0.001*** | -0.517 | -1.101 |  |  | Fetus | 1.758 | 0.473 | ***<0.001*** | 2.713 | 0.803 |
| 1 Day | -0.072 | 0.132 | 0.589 | 0.195 | -0.339 |  |  | 1 Day | 0.157 | 0.432 | 0.718 | 1.029 | -0.715 |
| 2 Weeks | 0.236 | 0.162 | 0.151 | 0.563 | -0.090 |  |  | 2 Weeks | -0.536 | 0.529 | 0.317 | 0.532 | -1.604 |
| 3-4 Years | 0 | 0.114 | 1 | 0.231 | -0.231 |  |  | 3-4 Years | 0 | 0.374 | 1 | 0.755 | -0.755 |
| 5-6 Years | -0.159 | 0.162 | 0.333 | 0.168 | -0.485 |  |  | 5-6 Years | 0.423 | 0.529 | 0.429 | 1.491 | -0.645 |
| 7-8 Years | -0.322 | 0.162 | 0.054 | 0.005 | -0.648 |  |  | 7-8 Years | 0.138 | 0.529 | 0.795 | 1.206 | -0.930 |
| 9-12 Years | -0.112 | 0.132 | 0.403 | 0.155 | -0.378 |  |  | 9-12 Years | -0.161 | 0.432 | 0.711 | 0.711 | -1.033 |
| 13-15 Years | 0.056 | 0.145 | 0.701 | 0.348 | -0.236 |  |  | 13-15 Years | -0.139 | 0.473 | 0.770 | 0.816 | -1.095 |
| 17-20 Years | -0.109 | 0.145 | 0.456 | 0.183 | -0.401 |  |  | 17-20 Years | -0.182 | 0.473 | 0.702 | 0.773 | -1.137 |
| 21-22 Years | -0.144 | 0.162 | 0.378 | 0.183 | -0.471 |  |  | 21-22 Years | -0.588 | 0.529 | 0.273 | 0.480 | -1.656 |
| ***App_751_*** | | | | | |  |  | ***App_ALL_*** | | | | | |
|  | β-coefficient | SE | *p*-value | 95% CI upper | 95% CI lower |  |  |  | β-coefficient | SE | *p*-value | 95% CI upper | 95% CI lower |
| Fetus | 0.699 | 0.106 | ***<0.001*** | 0.914 | 0.484 |  |  | Fetus | -0.068 | 0.069 | 0.331 | 0.072 | -0.207 |
| 1 Day | -1.014 | 0.097 | ***<0.001*** | -0.818 | -1.210 |  |  | 1 Day | -0.239 | 0.063 | ***<0.001*** | -0.112 | -0.366 |
| 2 Weeks | -0.642 | 0.119 | ***<0.001*** | -0.402 | -0.882 |  |  | 2 Weeks | 0.002 | 0.077 | 0.983 | 0.158 | -0.154 |
| 3-4 Years | 0 | 0.084 | 1 | 0.170 | -0.170 |  |  | 3-4 Years | 0 | 0.055 | 1 | 0.110 | -0.110 |
| 5-6 Years | 0.134 | 0.119 | 0.266 | 0.375 | -0.106 |  |  | 5-6 Years | -0.037 | 0.077 | 0.631 | 0.118 | -0.193 |
| 7-8 Years | -0.032 | 0.119 | 0.789 | 0.208 | -0.272 |  |  | 7-8 Years | -0.085 | 0.077 | 0.279 | 0.071 | -0.240 |
| 9-12 Years | 0.079 | 0.097 | 0.422 | 0.275 | -0.117 |  |  | 9-12 Years | 0.026 | 0.063 | 0.684 | 0.153 | -0.101 |
| 13-15 Years | -0.005 | 0.106 | 0.963 | 0.210 | -0.220 |  |  | 13-15 Years | -0.060 | 0.069 | 0.386 | 0.079 | -0.200 |
| 17-20 Years | -0.014 | 0.106 | 0.898 | 0.201 | -0.229 |  |  | 17-20 Years | -0.206 | 0.069 | *0.005* | -0.067 | -0.346 |
| 21-22 Years | -0.091 | 0.119 | 0.450 | 0.149 | -0.331 |  |  | 21-22 Years | -0.045 | 0.077 | 0.564 | 0.111 | -0.201 |
| ***Aqp4-M1*** | | | | | |  |  | ***Aqp4-M23*** | | | | | |
|  | β-coefficient | SE | *p*-value | 95% CI upper | 95% CI lower |  |  |  | β-coefficient | SE | *p*-value | 95% CI upper | 95% CI lower |
| Fetus | -1.941 | 0.227 | ***<0.001*** | -1.482 | -2.401 |  |  | Fetus | 0.662 | 0.227 | *0.006* | 1.120 | 0.203 |
| 1 Day | 0.856 | 0.208 | ***<0.001*** | 1.275 | 0.437 |  |  | 1 Day | 1.273 | 0.207 | ***<0.001*** | 1.691 | 0.855 |
| 2 Weeks | 0.425 | 0.254 | 0.102 | 0.938 | -0.088 |  |  | 2 Weeks | 0.866 | 0.254 | ***0.001*** | 1.378 | 0.353 |
| 3-4 Years | 0 | 0.180 | 1 | 0.363 | -0.363 |  |  | 3-4 Years | 0 | 0.179 | 1 | 0.362 | -0.362 |
| 5-6 Years | 0.258 | 0.254 | 0.316 | 0.772 | -0.255 |  |  | 5-6 Years | 0.249 | 0.254 | 0.332 | 0.761 | -0.263 |
| 7-8 Years | 0.480 | 0.254 | 0.066 | 0.993 | -0.033 |  |  | 7-8 Years | 0.383 | 0.254 | 0.139 | 0.896 | -0.129 |
| 9-12 Years | -0.120 | 0.208 | 0.566 | 0.299 | -0.539 |  |  | 9-12 Years | -0.090 | 0.207 | 0.665 | 0.328 | -0.509 |
| 13-15 Years | -0.030 | 0.227 | 0.896 | 0.429 | -0.489 |  |  | 13-15 Years | 0.065 | 0.227 | 0.775 | 0.524 | -0.393 |
| 17-20 Years | -0.118 | 0.227 | 0.608 | 0.342 | -0.577 |  |  | 17-20 Years | 0.040 | 0.227 | 0.862 | 0.498 | -0.419 |
| 21-22 Years | 0.334 | 0.254 | 0.196 | 0.847 | -0.179 |  |  | 21-22 Years | 0.442 | 0.254 | 0.089 | 0.954 | -0.070 |

**Supplementary Table S6: (Continued)**

| ***Gfapα*** | | | | | |  |  | ***Gfapδ*** | | | | | |
| --- | --- | --- | --- | --- | --- | --- | --- | --- | --- | --- | --- | --- | --- |
|  | β-coefficient | SE | *p*-value | 95% CI upper | 95% CI lower |  |  |  | β-coefficient | SE | *p*-value | 95% CI upper | 95% CI lower |
| Fetus | -2.393 | 0.158 | ***<0.001*** | -2.074 | -2.711 |  |  | Fetus | -1.563 | 0.271 | ***<0.001*** | -1.015 | -2.110 |
| 1 Day | 0.021 | 0.144 | 0.885 | 0.312 | -0.270 |  |  | 1 Day | -0.977 | 0.247 | ***<0.001*** | -0.478 | -1.476 |
| 2 Weeks | -0.137 | 0.176 | 0.441 | 0.219 | -0.493 |  |  | 2 Weeks | -1.176 | 0.303 | ***<0.001*** | -0.565 | -1.788 |
| 3-4 Years | 0 | 0.125 | 1 | 0.252 | -0.252 |  |  | 3-4 Years | 0.000 | 0.214 | 1.000 | 0.432 | -0.432 |
| 5-6 Years | -0.054 | 0.176 | 0.763 | 0.303 | -0.410 |  |  | 5-6 Years | -0.130 | 0.303 | 0.671 | 0.482 | -0.741 |
| 7-8 Years | -0.267 | 0.176 | 0.138 | 0.089 | -0.623 |  |  | 7-8 Years | -0.245 | 0.303 | 0.424 | 0.367 | -0.856 |
| 9-12 Years | -0.114 | 0.144 | 0.432 | 0.177 | -0.405 |  |  | 9-12 Years | -0.145 | 0.247 | 0.562 | 0.355 | -0.644 |
| 13-15 Years | -0.070 | 0.158 | 0.659 | 0.248 | -0.389 |  |  | 13-15 Years | -0.130 | 0.271 | 0.633 | 0.417 | -0.677 |
| 17-20 Years | 0.267 | 0.158 | 0.098 | 0.586 | -0.052 |  |  | 17-20 Years | 0.332 | 0.271 | 0.228 | 0.879 | -0.215 |
| 21-22 Years | 0.050 | 0.176 | 0.777 | 0.406 | -0.306 |  |  | 21-22 Years | -0.024 | 0.303 | 0.937 | 0.588 | -0.636 |
| ***Mapt-3R*** | | | | | |  |  | ***Mapt-4R*** | | | | | |
|  | β-coefficient | SE | *p*-value | 95% CI upper | 95% CI lower |  |  |  | β-coefficient | SE | *p*-value | 95% CI upper | 95% CI lower |
| Fetus | -1.096 | 0.184 | ***<0.001*** | -0.725 | -1.467 |  |  | Fetus | -4.661 | 0.131 | ***<0.001*** | -4.395 | -4.926 |
| 1 Day | 1.503 | 0.168 | ***<0.001*** | 1.842 | 1.165 |  |  | 1 Day | -0.846 | 0.120 | ***<0.001*** | -0.603 | -1.088 |
| 2 Weeks | 1.004 | 0.205 | ***<0.001*** | 1.419 | 0.589 |  |  | 2 Weeks | -0.466 | 0.147 | *0.003* | -0.169 | -0.762 |
| 3-4 Years | 0 | 0.145 | 1 | 0.293 | -0.293 |  |  | 3-4 Years | 0.000 | 0.104 | 1.000 | 0.210 | -0.210 |
| 5-6 Years | -0.162 | 0.205 | 0.436 | 0.253 | -0.577 |  |  | 5-6 Years | -0.140 | 0.147 | 0.347 | 0.157 | -0.436 |
| 7-8 Years | -0.239 | 0.205 | 0.251 | 0.176 | -0.654 |  |  | 7-8 Years | -0.144 | 0.147 | 0.333 | 0.153 | -0.441 |
| 9-12 Years | 0.038 | 0.168 | 0.821 | 0.377 | -0.301 |  |  | 9-12 Years | 0.083 | 0.120 | 0.492 | 0.325 | -0.159 |
| 13-15 Years | 0.026 | 0.184 | 0.888 | 0.397 | -0.345 |  |  | 13-15 Years | -0.035 | 0.131 | 0.793 | 0.231 | -0.300 |
| 17-20 Years | -0.282 | 0.184 | 0.132 | 0.089 | -0.653 |  |  | 17-20 Years | -0.264 | 0.131 | 0.051 | 0.001 | -0.530 |
| 21-22 Years | -0.171 | 0.205 | 0.411 | 0.244 | -0.586 |  |  | 21-22 Years | -0.190 | 0.147 | 0.204 | 0.107 | -0.486 |
| ***mKlotho*** | | | | | |  |  | ***sKlotho*** | | | | | |
|  | β-coefficient | SE | *p*-value | 95% CI upper | 95% CI lower |  |  |  | β-coefficient | SE | *p*-value | 95% CI upper | 95% CI lower |
| Fetus | -0.323 | 0.199 | 0.112 | 0.078 | -0.724 |  |  | Fetus | 1.431 | 0.215 | ***<0.001*** | 1.865 | 0.998 |
| 1 Day | -0.482 | 0.181 | *0.011* | -0.116 | -0.848 |  |  | 1 Day | 0.461 | 0.196 | *0.024* | 0.858 | 0.065 |
| 2 Weeks | -0.225 | 0.222 | 0.317 | 0.224 | -0.673 |  |  | 2 Weeks | 0.114 | 0.240 | 0.637 | 0.599 | -0.371 |
| 3-4 Years | 0 | 0.157 | 1 | 0.317 | -0.317 |  |  | 3-4 Years | 0 | 0.170 | 1 | 0.343 | -0.343 |
| 5-6 Years | -0.140 | 0.222 | 0.531 | 0.308 | -0.589 |  |  | 5-6 Years | -0.147 | 0.240 | 0.544 | 0.338 | -0.632 |
| 7-8 Years | -0.221 | 0.222 | 0.325 | 0.227 | -0.670 |  |  | 7-8 Years | 0.053 | 0.240 | 0.825 | 0.538 | -0.432 |
| 9-12 Years | 0.306 | 0.181 | 0.099 | 0.672 | -0.060 |  |  | 9-12 Years | 0.042 | 0.196 | 0.833 | 0.438 | -0.355 |
| 13-15 Years | 0.247 | 0.199 | 0.221 | 0.648 | -0.154 |  |  | 13-15 Years | -0.018 | 0.215 | 0.933 | 0.416 | -0.452 |
| 17-20 Years | 0.245 | 0.199 | 0.224 | 0.646 | -0.156 |  |  | 17-20 Years | 0.070 | 0.215 | 0.745 | 0.504 | -0.364 |
| 21-22 Years | -0.110 | 0.222 | 0.624 | 0.339 | -0.558 |  |  | 21-22 Years | 0.049 | 0.240 | 0.841 | 0.534 | -0.437 |

**Supplementary Table S7: Senescence-related isoform expression levels in naked mole-rat brain across a 22-year time series**

Senescence-related isoform expression levels in brain tissue of naked mole-rats from fetus through to 22-year-old, stratified into age groups, by linear regression. Transcripts showing nominal associations (p<0.05) are shown in italic and underlined, those which meet FDR correction for multiple testing (p<0.00195) are shown in bold italic and underlined. SE: standard error, 95% CI: 95% confidence intervals.

| ***Atm*** | | | | | |  |  | ***Cdkn1a (p21a)*** | | | | | |
| --- | --- | --- | --- | --- | --- | --- | --- | --- | --- | --- | --- | --- | --- |
|  | β-coefficient | SE | *p*-value | 95% CI upper | 95% CI lower |  |  |  | β-coefficient | SE | *p*-value | 95% CI upper | 95% CI lower |
| Fetus | 0.883 | 0.264 | ***0.002*** | 1.417 | 0.350 |  |  | Fetus | 1.040 | 0.259 | ***<0.001*** | 1.563 | 0.518 |
| 1 Day | 0.310 | 0.241 | 0.205 | 0.797 | -0.177 |  |  | 1 Day | 0.097 | 0.236 | 0.683 | 0.574 | -0.380 |
| 2 Weeks | 0.180 | 0.295 | 0.544 | 0.776 | -0.416 |  |  | 2 Weeks | 0.782 | 0.289 | *0.010* | 1.366 | 0.198 |
| 3-4 Years | 0 | 0.209 | 1 | 0.421 | -0.421 |  |  | 3-4 Years | 0 | 0.204 | 1 | 0.413 | -0.413 |
| 5-6 Years | 0.263 | 0.295 | 0.377 | 0.860 | -0.333 |  |  | 5-6 Years | -0.125 | 0.289 | 0.669 | 0.459 | -0.709 |
| 7-8 Years | 0.558 | 0.295 | 0.066 | 1.154 | -0.038 |  |  | 7-8 Years | -0.019 | 0.289 | 0.949 | 0.565 | -0.603 |
| 9-12 Years | -0.181 | 0.241 | 0.456 | 0.305 | -0.668 |  |  | 9-12 Years | 0.230 | 0.236 | 0.335 | 0.707 | -0.247 |
| 13-15 Years | -0.039 | 0.264 | 0.882 | 0.494 | -0.573 |  |  | 13-15 Years | -0.027 | 0.259 | 0.916 | 0.495 | -0.550 |
| 17-20 Years | -0.179 | 0.264 | 0.501 | 0.354 | -0.712 |  |  | 17-20 Years | 0.115 | 0.259 | 0.658 | 0.638 | -0.407 |
| 21-22 Years | 0.206 | 0.295 | 0.488 | 0.802 | -0.390 |  |  | 21-22 Years | 0.014 | 0.289 | 0.961 | 0.598 | -0.570 |
| ***Cdkn2a (p14^ARF^)*** | | | | | |  |  | ***Cdkn2a (p16^INK4a^)*** | | | | | |
|  | β-coefficient | SE | *p*-value | 95% CI upper | 95% CI lower |  |  |  | β-coefficient | SE | *p*-value | 95% CI upper | 95% CI lower |
| Fetus | -2.414 | 0.175 | ***<0.001*** | -2.060 | -2.768 |  |  | Fetus | -3.472 | 0.189 | ***<0.001*** | -3.090 | -3.854 |
| 1 Day | -3.092 | 0.196 | ***<0.001*** | -2.696 | -3.488 |  |  | 1 Day | -4.699 | 0.243 | ***<0.001*** | -4.206 | -5.193 |
| 2 Weeks | -2.965 | 0.196 | ***<0.001*** | -2.569 | -3.361 |  |  | 2 Weeks | -4.349 | 0.298 | ***<0.001*** | -3.745 | -4.954 |
| 3-4 Years | 0 | 0.138 | 1 | 0.280 | -0.280 |  |  | 3-4 Years | 0 | 0.149 | 1 | 0.302 | -0.302 |
| 5-6 Years | 0.118 | 0.196 | 0.549 | 0.514 | -0.278 |  |  | 5-6 Years | 0.123 | 0.211 | 0.564 | 0.550 | -0.305 |
| 7-8 Years | 0.345 | 0.196 | 0.086 | 0.741 | -0.051 |  |  | 7-8 Years | 0.307 | 0.211 | 0.154 | 0.735 | -0.120 |
| 9-12 Years | 0.324 | 0.160 | *0.050* | 0.647 | 0.001 |  |  | 9-12 Years | 0.504 | 0.172 | *0.006* | 0.853 | 0.155 |
| 13-15 Years | 0.079 | 0.175 | 0.656 | 0.433 | -0.276 |  |  | 13-15 Years | 0.263 | 0.189 | 0.172 | 0.645 | -0.120 |
| 17-20 Years | 0.290 | 0.175 | 0.106 | 0.644 | -0.064 |  |  | 17-20 Years | 0.308 | 0.189 | 0.112 | 0.690 | -0.075 |
| 21-22 Years | 0.302 | 0.196 | 0.131 | 0.698 | -0.094 |  |  | 21-22 Years | 0.484 | 0.211 | *0.028* | 0.912 | 0.057 |
| ***Cdkn2b (p15^INK4b^)*** | | | | | |  |  | ***Tp53*** | | | | | |
|  | β-coefficient | SE | *p*-value | 95% CI upper | 95% CI lower |  |  |  | β-coefficient | SE | *p*-value | 95% CI upper | 95% CI lower |
| Fetus | -0.336 | 0.144 | *0.024* | -0.046 | -0.626 |  |  | Fetus | 3.111 | 0.131 | ***<0.001*** | 3.375 | 2.847 |
| 1 Day | -1.056 | 0.131 | ***<0.001*** | -0.791 | -1.321 |  |  | 1 Day | 0.295 | 0.119 | *0.018* | 0.536 | 0.054 |
| 2 Weeks | -0.446 | 0.160 | *0.008* | -0.122 | -0.771 |  |  | 2 Weeks | 0.283 | 0.146 | 0.060 | 0.578 | -0.012 |
| 3-4 Years | 0 | 0.113 | 1 | 0.229 | -0.229 |  |  | 3-4 Years | 0 | 0.103 | 1 | 0.209 | -0.209 |
| 5-6 Years | -0.176 | 0.160 | 0.278 | 0.148 | -0.500 |  |  | 5-6 Years | -0.004 | 0.146 | 0.977 | 0.291 | -0.299 |
| 7-8 Years | -0.176 | 0.160 | 0.279 | 0.148 | -0.500 |  |  | 7-8 Years | -0.117 | 0.146 | 0.428 | 0.178 | -0.412 |
| 9-12 Years | 0.172 | 0.131 | 0.196 | 0.437 | -0.093 |  |  | 9-12 Years | 0.081 | 0.119 | 0.502 | 0.322 | -0.160 |
| 13-15 Years | 0.101 | 0.144 | 0.486 | 0.391 | -0.189 |  |  | 13-15 Years | 0.069 | 0.131 | 0.599 | 0.333 | -0.195 |
| 17-20 Years | 0.179 | 0.144 | 0.219 | 0.469 | -0.111 |  |  | 17-20 Years | 0.081 | 0.131 | 0.537 | 0.345 | -0.183 |
| 21-22 Years | 0.014 | 0.160 | 0.929 | 0.338 | -0.310 |  |  | 21-22 Years | -0.112 | 0.146 | 0.448 | 0.183 | -0.407 |
